# Identification of basement membrane-related prognostic model associated with the immune microenvironment and synthetic therapy response in pancreatic cancer: integrated bioinformatics analysis and clinical validation

**DOI:** 10.7150/jca.100891

**Published:** 2024-10-14

**Authors:** Biao Zhang, Xu Chen, Huiyi Song, Xue Gao, Shurong Ma, Hongying Ji, Huixian Qu, Shilin Xia, Dong Shang

**Affiliations:** 1 Pancreas & Biliary Center, Department of General Surgery, Clinical Laboratory of Integrative Medicine, The First Affiliated Hospital of Dalian Medical University, Dalian, China.; 2 Institute (College) of Integrative Medicine, Dalian Medical University, Dalian, China.; 3 Department of Pathology, The First Affiliated Hospital of Dalian Medical University, Dalian, China.

**Keywords:** Pancreatic cancer, Basement membrane, Prognosis, Tumor immune microenvironment, Therapy

## Abstract

Pancreatic cancer (PC) is a common and highly malignant tumor. Basement membrane (BM) is formed by the crosslinking of extracellular matrix macromolecules and acts as a barrier against tumor cell metastasis. However, the role of BM in PC prognosis, immune infiltration, and treatment remains unclear. This study collected transcriptome and clinical survival data of PC via TCGA, GEO, and ICGC databases. PC patients (PCs) from the First Affiliated Hospital of Dalian Medical University were obtained as the clinical validation cohort. BM-related genes (BMRGs) were acquired from GeneCards and basement membraneBASE databases. A total of 46 differential-expressed BMRGs were identified. Then the BM-related prognostic model (including DSG3, MET, and PLAU) was built and validated. PCs with a low BM-related score had a better outcome and were more likely to benefit from oxaliplatin, irinotecan, and KRAS(G12C) inhibitor-12, and immunotherapy. Immune analysis revealed that BM-related score was positively correlated with neutrophils, cancer-associated fibroblasts, and macrophages infiltration, but negatively correlated with CD8+ T cells, NK cells, and B cells infiltration. PCs from the clinical cohort further verified that BM-related model could accurately predict PCs' outcomes. DSG3, MET, and PLAU were notably up-regulated within PC tissues and linked to a poor prognosis. *In vitro* experiments showed that DSG3 knockdown markedly suppressed the proliferation, migration, and invasion of PC cells. Molecular docking indicated that epigallocatechin gallate had a strong binding activity with DSG3, MET, and PLAU and may be used as a potential therapeutic agent for PC. In conclusion, this study developed a BM-related model associated with PC prognosis, immune infiltration, and treatment, which provided new insights into PC stratification and drug intervention.

## 1. Introduction

Pancreatic cancer (PC) has an occult onset, aggressive spread, and extremely poor prognosis. PC will likely become the major factor of cancer-related mortality because of its rising worldwide incidence in recent years. In 1990, there were about 196,000 new diagnoses of PC worldwide, but this increased to 441,000 cases in 2017^1^. Worldwide, PC was the fourth most prevalent malignant tumor and the seventh-highest rate of death due to cancer^2^. In America, PC was the ninth most prevalent malignant neoplasm and the third-highest rate of death due to cancer^3^, and was projected to become the second-highest rate of death due to cancer after lung carcinoma in 2030^4^. In China, PC ranked as the tenth most prevalent malignant neoplasm and the sixth-highest rate of death due to cancer^5^. Due to the concealed beginning and the dearth of reliable early detection techniques, most patients have been diagnosed with unresectable or metastatic PC, only 15%-20% are suitable for surgical resection^6^. Chemotherapy is the primary treatment for PC besides surgery, and first-line regimens include FOLFIRINOX (fluorouracil, oxaliplatin, irinotecan, and leucovorin) and gemcitabine in combination with albumin-paclitaxel^7^. A study showed that the median survival (MS) of advanced PC that received the FOLFIRINOX regimen or gemcitabine was only 11.1 months or 6.8 months^8^. The MS of resected PC that received modified FOLFIRINOX or gemcitabine was only 54.4 months or 35.0 months^9^. Recently, targeted therapy and immunotherapy have continued to make advances and achieved good efficacy in some tumors, bringing new hope to tumor patients. However, they did not exhibit the expected efficacy in PC, and most of them failed in phase 1 and 2 clinical trials^10^. A study indicated that ibrutinib and durvalumab for treating PC had a complete or partial response rate of only 2%^11^. Thus, it is critical to investigate the mechanisms of PC development, create effective early screening techniques, and find new treatment strategies.

Invasion and metastasis are important features of malignant tumors, and about 66.7% of patients with solid tumors die from metastasis^12^. The process of cancer cell invasion and metastasis needs destruction and penetration of the basement membrane (BM). BM is a special barrier formed by the cross-linking of macromolecules (laminin, type IV collagen, nidogen, proteoglycan, etc.) in extracellular matrix (ECM), with a dense structure and a pore size of about 50nm. BM is located beneath epithelia and endothelia and is crucial for cell adhesion, migration, and survival^13^-^16^. Several studies have been conducted to explore how cancer cells cross BM. Cancer cells themselves can form invadopodia rich in proteases and actin. Invadopodia can degrade BM components through proteases or reshape BM through mechanical force to form micron-sized pores, thereby mediating cancer cells to penetrate BM^17^,^18^. Glentis *et al.*^19^ found that cancer-associated fibroblasts (CAFs) could widen and intenerate BM channels that allow tumor cells to spread.

The abnormality of BM is linked to the onset, development, and outcome of numerous illnesses, especially malignant tumors^20^-^22^. Studies indicated that the degree of BM cross-linking was significantly reduced in malignant tumors, and defective or disrupted BM was linked to tumor development and unfavorable prognosis^23^,^24^. Pathological examination revealed that the defect of BM occurred during the development of preinvasive intraductal cancer into invasive breast cancer (BC)^25^. BC patients had a five-year survival rate of up to 99% if the cancer cells are limited to BM; 85% if the cancer cells penetrate BM and invade surrounding tissues; and just 27% if long-distance metastases take place^16^. When bladder cancer does not break through BM and lamina propria, the five-year survival rate can reach 80%, whereas only 20% when it spreads to the bladder wall's entire thickness^26^. Van der Zee *et al.*^27^ found that in PC, laminin was strongly linked to postoperative survival, and type IV collagen was highly related to pathological grade. In addition, dysregulated BM is also closely related to tumor immunity and therapeutic response. A study showed that laminin in BM can participate in regulating the activation, function, and multiplication of T cells^28^. Therefore, exploring the process and mechanism of tumor cells overcoming the BM barrier can offer novel strategies for PC therapy.

Based on the above evidence, this study utilized multiple public datasets and clinical cohort to build and validate the BM-derived model associated with PC prognosis, immune microenvironment, and synthetic therapy response. Besides, we identified that DSG3, MET, and PLAU were notably up-regulated within PC tissues and related to a bad outcome. The knockdown of DSG3 could inhibit the proliferation, migration, and invasion of PC cells. Epigallocatechin gallate had a strong binding activity with DSG3, MET, and PLAU and may be used as a potential therapeutic agent for PC.

## 2. Materials and methods

### 2.1 Data collection and preprocessing

We collected the publicly available PC cohorts, which includes PC patients with transcriptomic and clinical survival data. Transcript, single nucleotide variation (SNV), copy number variation (CNV), and clinical survival information (comprising 185 PCs, **Table S1**) were downloaded via The Cancer Genome Atlas (TCGA) platform (https://portal.gdc.cancer.gov/). PACA-AU cohort (comprising 81 PCs) was obtained through International Cancer Genome Consortium (ICGC) platform (https://dcc.icgc.org/releases/current/Projects). GSE28735 (comprising 45 pairs of PC and normal tissues), GSE62452 (comprising 69 PC tissues and 61 normal tissues), GSE57495 (comprising 63 PC tissues), GSE78229 (comprising 50 PC tissues), and GSE85916 (comprising 80 PC tissues) datasets were collected through Gene Expression Omnibus (GEO) platform (https://www.ncbi.nlm.nih.gov/geo/). We employed “sva” tool to get rid of the batch effect among various datasets^29^. For screening the differentially expressed genes (DEGs) in PC and normal tissues, Genotype-Tissue Expression Project (GTEx) dataset (comprising 167 healthy human pancreatic tissues) and TCGA dataset (comprising 178 PC tissues and 4 normal tissues) were collected through UCSC Xena platform (https://xenabrowser.net/datapages/).

### 2.2 Identification and analysis of differentially-expressed BMRGs

DEGs were recognized in PC and normal tissues by employing the “limma” package. |log2 fold variance (FC)| > 1 and an adjusted p-value < 0.05 were the selection parameters^30^. BMRGs were collected via GeneCards platform (https://www.genecards.org/) as well as basement membraneBASE platform (https://bmbase.manchester.ac.uk)^31^. DEGs and BMRGs were intersected to obtain differentially expressed BMRGs. The “maftools” and “RCircos” packages were applied to investigate genetic alterations of differentially expressed BMRGs in PC. GO and KEGG enrichment analyses were employed to seek the biological roles involved in differentially expressed BMRGs.

### 2.3 Building BM-related prognostic model

According to PC sample ID, the expression and survival data were merged. TCGA samples were split 7:3 at random into training and testing sets utilizing “caret” package^32^. Samples from ICGC-PACA-AU, GSE28735, GSE62452, GSE57495, GSE78229, as well as GSE85916 cohorts, served as verification from outside. Initially, prognosis-related BMRGs were screened utilizing univariable Cox regression. Then, LASSO regression was performed to combat overfitting. Finally, multivariable Cox regression was employed to further screen prognosis-related BMRGs and establish BM-related model. BM-related risk score of each sample could be computed through “predict” function. The risk score's median value in the training cohort was applied to categorize each sample as either a high-risk or low-risk group. Kaplan-Meier (KM) curves were employed to contrast the survival periods. Receiver Operating Characteristic (ROC) curves with a time dependence were applied to assess the model's capability for prediction. In addition, we searched twelve previously published studies on prognostic models in PC, and relevant genes were obtained from published prognostic models^33^-^44^. ROC curves and C-index were calculated to compare the predictive performance of BM-related model with published prognostic models in PC.

### 2.4 Clinicopathological characteristics correlation and nomogram model building

PCs were grouped by their clinicopathological features, and risk score in various subgroups was contrasted. Cox regression analysis was implemented to seek the independent prognostic variables. Clinicopathological features as well as risk score were further employed to build a nomogram model utilizing “rms” package. The calibration curve was used to evaluate the predictive performance.

### 2.5 Gene set enrichment analysis (GSEA)

GSEA was implemented to investigate the potential differences in metabolic processes and biological behaviours across various subgroups. And the reference gene sets “c5.go.v7.5.1.symbols.gmt” and “c2. cp.kegg.v7.5.1.symbols.gmt” were downloaded via MSigDB platform (https://www.gsea-msigdb.org/gsea/msigdb/)^45^.

### 2.6 Immunoassay

Single-sample GSEA (ssGSEA) was implemented to quantify the score of 16 immune cells and 13 immune-linked pathways in various subgroups. In addition, the infiltration scores of immune cell subpopulations calculated through multiple algorithms for TCGA tumors were downloaded via Tumor Immune Estimation Resource database (TIMER, http://timer.cistrome.org/)^46^. A platform created around TCGA database is known as The Cancer Immunome Atlas (TCIA, https://tcia.at/)^47^. It could investigate the intra-tumoral microenvironment and antigenic gene of 20 types of solid tumors, along with evaluating immune phenotype score (IPS) and forecast the susceptibility of CTLA-4 and PD-1 blockers. Therefore, the IPS of PCs in the TCGA cohort was acquired via TCIA platform. Tumor immune dysfunction and exclusion (TIDE) platform (http://tide.dfci.harvard.edu/) could assess the sensitivity of immunotherapy by mimicking tumor immune escape mechanism and was used to validate the relationship between BM-related score and immunotherapy^48^. The IMvigor210 cohort was an immunotherapy cohort research for urothelial carcinoma (UC) and was obtained in this study (http://research-pub.gene.com/IMvigor210CoreBiologies)^49^. Risk scores for UC patients were figured out based on BM-related prognostic model. Then, the relationship between BM-related risk scores and immunotherapy was evaluated.

### 2.7 Drug sensitivity prediction

The “oncoPredict” is an R package with the ability to forecast therapy responses and biomarkers for cancer patients based on cell line filtrating data^50^. Therefore, the “oncoPredict” package was employed in this study to assess variations in drug sensitivity across different PC subgroups.

### 2.8 Acquisition of PC tissues

We obtained 10 pairs of PC and normal tissues, which were derived from those with PC undergoing surgery in the First Affiliated Hospital of Dalian Medical University during 2022.01.01-2024.08.15. All tissues were preserved at -80 degrees Celsius before RNA was extracted. In addition, we also obtained formalin-fixed paraffin-embedded pathological sections of 54 PC tissues and 22 normal tissues, which were derived from those with PC undergoing surgery in the First Affiliated Hospital of Dalian Medical University during 2016.01.01 and 2021.12.31. All paraffin slices were stained utilizing hematoxylin and eosin methods, and PC was diagnosed through two experienced pathologists. The clinical stage and pathological grade of 54 PCs were obtained through the medical record system in our hospital. Survival data was collected via telephone follow-up until 2023.03.28 **(Table S2)**. Among the 54 PCs, two patients died from postoperative complications, and two failed for follow-up. Therefore, these four patients were not included in the prognostic analysis.

### 2.9 Immunohistochemistry

Immunohistochemistry was implemented for the pathological sections of 54 PC tissues and 22 paracancerous tissues. The paraffin-embedded pathological specimens were split with an average thickness of 3 μm in a serial fashion and placed onto glass slides. Bake the slides in an oven at 60 ℃ for 1 hour, then dewax until hydrated. Place the sliced tissue into EDTA antigen retrieval solution (PH 9.0), and perform antigen retrieval under the conditions of microwave medium heat for 8 minutes, cease fire for 8 minutes, and medium-low heat for 7 minutes. Use 3% hydrogen peroxide solution to incubate within darkness at ambient temperature for 25 minutes to block endogenous peroxidase. After that, wash the sectioned tissue three times in PBS for 5 minutes on each occasion. Add 3% bovine serum albumin (BSA) dropwise to evenly cover the sliced tissues, and block for 30 minutes. Mouse anti-human DSG3 monoclonal antibody (R&D Systems, MAB1720, concentration 8ug/mL), rabbit anti-human MET monoclonal antibody (Abcam, ab216574, dilution 1:1000), and rabbit anti-human PLAU polyclonal antibody (Abcam, ab24121, dilution 1:200) were used to be the initial antibodies and incubated at 4 ℃ for a whole night. Add a secondary antibody (horseradish peroxidase label) that match the initial antibody species and incubate at ambient temperature for 50 minutes. Use freshly prepared 3.3'-diaminobenzidine for color development. Sliced tissues were counterstained with hematoxylin and finally dehydrated and cleared using ethanol and xylene. The sections were examined under light microscopy. The tumor areas and adjacent areas were confirmed by pathologists. Three typical representative fields of view were selected for each of the tumor areas and adjacent areas under a 200× microscope (OLYMPUS DP73) for image collection. Immunohistochemical images were quantitatively evaluated using ImageJ software. Positive signals were quantified as mean optical density values (integrated option density/area).

### 2.10 Immunofluorescence

Immunofluorescence was performed to investigate differences in BM structures between PC tissues and normal tissues. Paraffin-embedded pathological specimens were sectioned at a thickness of 3 µm and mounted onto slides. The slides were baked at 60°C for 1 hour and then deparaffinized and rehydrated. Antigen retrieval was performed using EDTA. The sliced tissues were then incubated in Triton X-100 solution for 45 minutes to enhance cell membrane permeability. Blocking was carried out with goat serum at room temperature for 1 hour. Subsequently, rabbit anti-human Collagen IV monoclonal antibody (Abcam, ab214417, dilution 1:500) were used to be the initial antibodies and incubated at 4 ℃ for a whole night. Then, secondary antibodies specific to the primary antibodies were added and incubated at room temperature for 1 hour. DAPI staining solution was used for nuclear counterstaining. Finally, images were captured using a Keyence BZ-X810 fluorescence microscope.

### 2.11 Cell culture and siRNA transfection

PC cell lines CFPAC-1 and BxPC-3 were acquired via Haixing Biosciences Co., Ltd, Suzhou, China. CFPAC-1 and BxPC-3 cells were cultured using Iscove's Modified Dulbecco's Medium and RPMI 1640 mixed with 10% fetal bovine serum (FBS, Gibco, USA), respectively. The incubation environment was 37 ° C with a humidity of 95% air and 5% CO^2^. Transfection reagent (Transfect-Mate) and siRNA were provided through GenmaPharma (Suzhou, China). The siRNA sequences targeting DSG3 are as below: siRNA#1: 5'-GUCCGUACUUUGACCAAUUTT-3'; siRNA#2: 5'-GGCUUGCAGUAUAUUUCUUTT-3'.

### 2.12 PCR

All the RNAs of PC cells and tissues were collected by an extraction tool named TRIzol (Adamas life, Titan Scientific Co., Ltd., Shanghai, China). The Reverse Transcription Reagent (Yugong Biolabs Co., Ltd., Jiangsu, China) was utilized to obtain cDNAs. Real-time quantitative PCR was implemented by utilizing PCR Kit (Yugong Biolabs Co., Ltd., Jiangsu, China). The control reference used in this study was GAPDH. The ΔΔCt technique was utilized to quantify the expressed level of RNA. The primer sequence for humans was acquired via Sangon Biotech (Shanghai, China), as below DSG3, 5'-GAACCAGCAGGCACACCCATG-3' (Forward), 5'-CACCACTCACAACCAGACGATAGC-3' (Reverse); MET, 5'-GTCCTATGGCTGGTGGCACTTTAC-3' (Forward), 5'-TGGTTTGGGCTGGGGTATAACATTC-3' (Reverse); PLAU, 5'-TCGCTCAAGGCTTAACTCCAACAC-3' (Forward), 5'-ACGGATCTTCAGCAAGGCAATGTC-3' (Reverse).

### 2.13 CCK-8 assay

CFPAC-1 and BxPC-3 cells were seeded in 96-well plates with 1 ⅹ 10^4^ cells per well, and each group had 5 secondary wells. Cell viability was measured at 0, 24, 48, and 72 hours, respectively. Add 100 ml of serum-free culture containing 10% CCK-8 enhanced solution to each well and incubate for 2 hours in a cell culture incubator. Then the OD value was measured at 450nm.

### 2.14 Scratch assay

The transfected CFPAC-1 and BxPC-3 cells were seeded in a 6-well plate. When the cell growth adhesion density reached 100%, a 200uL pipette tip was utilized to scratch the bottom of the plate vertically. Wash with PBS three times, and then take pictures at 0 and 24 hours. ImageJ software was utilzied to calculate the scratch area.

### 2.15 Transwell assay

A 24-well transwell chamber (Corning, NY, USA) was employed for Transwell invasion assay. The invasion assay required presetting Matrigel (Nova Medical Technology Co., Ltd., Shanghai, China) in transwell chamber. 1 ⅹ 10^5^ BxPC-3 cells or 2 ⅹ 10^5^ CFPAC-1 cells with 100uL serum-free culture were inoculated in the upper chamber, and 800uL culture containing 10% FBS was added to the lower chamber. The cells were counted after incubation for 24 hours.

### 2.16 Candidate drug prediction and molecular docking

Drug molecules that could target model genes were retrieved via the comparative toxicogenomics database (https://ctdbase.org/)^51^. The 2D structure of the drug molecule was downloaded via the PubChem database (https://pubchem.ncbi.nlm.nih.gov)^52^ and converted to 3D structure using Chem3D software to optimize the structure with minimum free energy. The 3D structure of DSG3, MET, and PLAU protein was downloaded from the Protein Data Bank (PDB) database (https://www.rcsb.org)^53^, and water and heterogeneous molecules were removed using PyMol software. The protein was hydrogenated using AutoDockTools, and the docking box parameters were set using the Gird module: Spacing (angstrom) equals 1. AutoDock vina was used to perform molecular docking between the drug molecule and the protein and calculate the binding energy. The smaller the binding energy, the more stable the docking. Binding energy less than -4.25 kcal/mol indicated the presence of binding activity, less than -5.0 kcal/mol indicated good binding activity, and less than -7.0 kcal/mol indicated strong docking activity^54^. The molecular docking results were visualized using PyMol software.

### 2.17 Data analysis

Data analysis and visualization were performed using R software (Ver 4.1.2) and GraphPad Prism 9. For continuous variables that conform to a normal distribution, t-test was used to compare differences between two groups. For continuous variables that do not conform to the normal distribution, Wilcoxon signed-rank test was used to compare differences between two groups, and Kruskal-Wallis test was used to compare differences among multiple groups. KM method was used to draw survival curves, and the log-rank test was used to compare the survival times of different groups. The p-value < 0.05 indicates statistical significance.

## 3. Results

### 3.1 Identification and analysis of differentially expressed BMRGs

**Figure 1:**
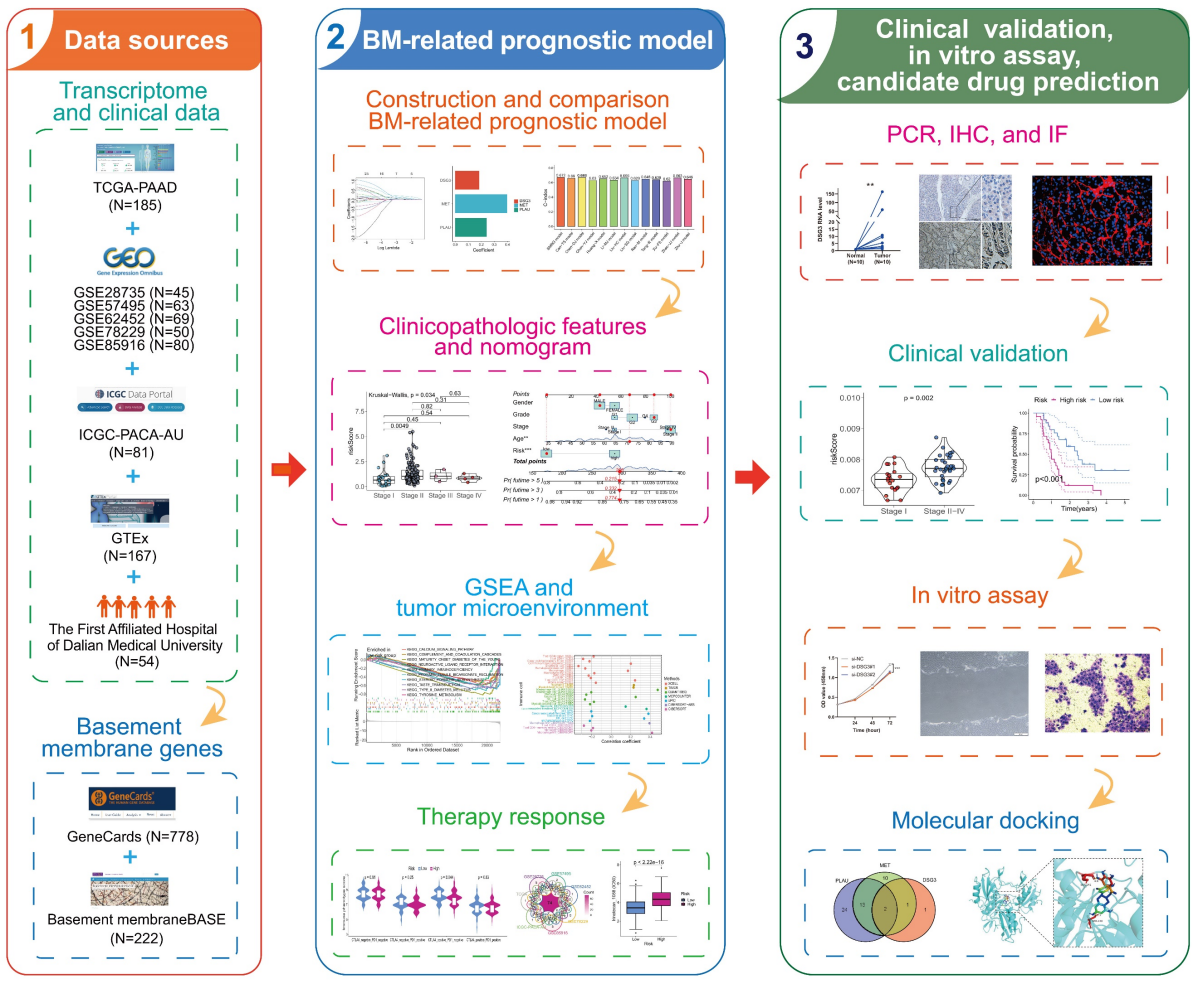
Flowchart of this study.

**Figure 2:**
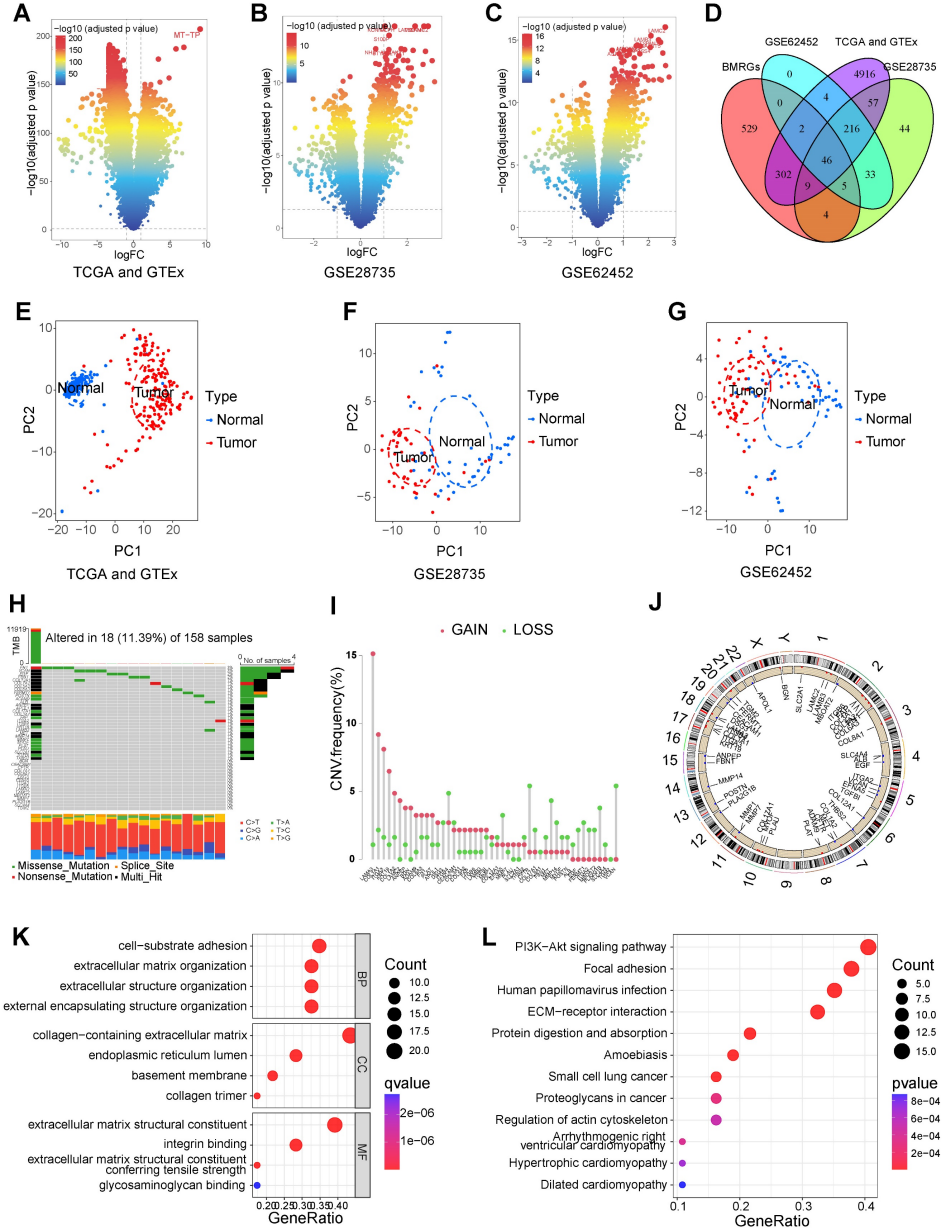
Identification and analysis of differentially-expressed basement membrane-related genes (BMRGs). The differentially expressed genes (DEGs) between pancreatic tumors and normal tissues from TCGA and GTEx datasets **(A)**, GSE28735 dataset **(B)**, and GSE62452 dataset **(C)**. **(D)** The intersection of DEGs and BMRGs. Principal component analysis based on the expression of differentially-expressed BMRGs could clearly distinguish pancreatic tumor and normal tissues in TCGA and GTEx **(E)**, GSE28735 **(F)**, and GSE62452 **(G)** datasets. **(H)** The SNV of differentially-expressed BMRGs in pancreatic cancer (PC). **(I)** Frequency of copy number variation (CNV) of BMRGs in PC. **(J)** The location on the chromosome where CNV occurs. GO **(K)** and KEGG **(L)** analyses.

The overall process of this study is shown in **Figure 1**. We identified 5552 DEGs between PC and normal tissues from TCGA and GTEx datasets (**Figure 2A**), 415 DEGs between PC and normal tissues from GSE28735 dataset (**Figure 2B**), and 306 DEGs between PC and normal tissues from GSE62452 dataset (**Figure 2C**). 778 BMRGs were obtained from the GeneCards database with the filter condition of relevance score > 5. 222 BMRGs were obtained from the basement membraneBASE platform. A total of 898 BMRGs were obtained after eliminating 102 repeated genes **(Table S3)**. Finally, 46 differentially expressed BMRGs were obtained by intersecting DEGs and BMRGs **(Figure 2D)**. Based on 46 differentially expressed BMRGs, principal component analysis (PCA) can clearly distinguish PC and normal tissues derived from TCGA and GTEx **(Figure 2E)**, GSE28735 **(Figure 2F)**, and GSE62452 **(Figure 2G)** datasets, suggesting that these BMRGs could be closely related to the occurrence of PC.

The genetic alterations of 46 differentially expressed BMRGs in PC were investigated. Results showed that the SNV frequency of 46 BMRGs in PC was relatively low. Among 158 samples, 18 cases (11.39%) had SNV. The top four genes with the highest SNV frequency were FN1 (3%), VCAN (3%), EGF (2%), and FBN1 (2%). The most common type of SNV is missense mutation **(Figure 2H)**. CNV occurred in 46 BMRGs, and the four genes with the highest frequency were LAMA3, COL1A1, IGTA3, and KRT19 **(Figure 2I)**. The location of CNV on the chromosome was exhibited in **Figure 2J**.

GO and KEGG enrichment analyses were implemented to investigate the biological functions and processes involved in 46 BMRGs. GO enrichment analysis showed that “cell-substrate adhesion”, “extracellular matrix organization”, “extracellular structure organization”, and “external encapsulating structure organization” were significantly enriched in biological process (BP); “collagen-containing extracellular matrix”, “endoplasmic reticulum lumen”, “basement membrane”, and “collagen trimer” were significantly enriched in cellular component (CC); “extracellular matrix structural constituent”, “integrin binding”, “extracellular matrix structural constituent conferring tensile strength”, and “glycosaminoglycan binding” were significantly enriched in molecular function (MF) **(Figure 2K)**. KEGG pathway enrichment analysis showed that “ECM-receptor interaction”, “focal adhesion”, “PI3K-Akt signaling pathway”, “small cell lung cancer”, and “proteoglycans in cancer” were significantly enriched **(Figure 2L)**.

### 3.2 Construction, validation, and comparison of BM-related prognostic model

While previous research has reported the clinical value of BM-related signatures in various human malignancies, including PC, these studies typically used only one or two publicly available PC cohorts^55^,^56^. Our study collected seven publicly available PC cohorts. More importantly, we further validate the model's reliability with a clinical cohort from the First Affiliated Hospital of Dalian Medical University.

**Figure 3:**
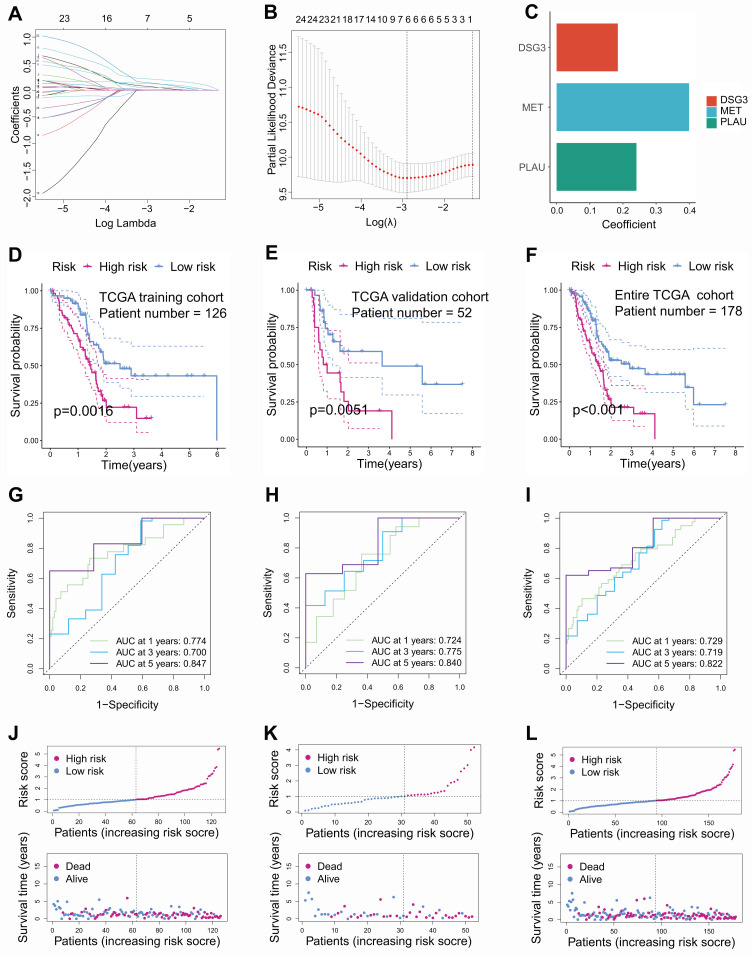
Construction and internal validation of basement membrane-related prognostic model. Coefficient path diagram **(A)** and cross-validation curve **(B)** of LASSO regression. **(C)** DSG3, MET, and PLAU were utilized to build the basement membrane-related prognostic model. Kaplan-Meier curves showed that the survival time of low-risk pancreatic cancer (PC) was notably higher than that of high-risk PC in the training cohort **(D)**, internal validation cohort **(E)**, and the entire TCGA dataset **(F)**. ROC curves of the training cohort **(G)**, internal validation cohort **(H)**, and the entire TCGA dataset **(I)**. Risk score curves and survival status scatter plots of the training cohort **(J)**, internal validation cohort **(K)**, and the entire TCGA dataset **(L)**.

In this study, PC samples from the TCGA database were randomly divided into a training cohort and an internal validation cohort according to 7:3. Univariable Cox regression identified 25 prognostic-related BMRGs **(Table S4)**. To prevent overfitting among genes, six prognostic-related BMRGs were screened using LASSO regression **(Figures 3A and B)**. Finally, multivariable Cox regression was utilized to further obtain prognostic-related BMRGs and build a prognostic model including DSG3, MET, and PLAU **(Figure 3C)**. KM curves showed that in the training cohort **(Figure 3D)**, internal validation cohort **(Figure 3E)**, and the entire TCGA cohort **(Figure 3F)**, the survival time of the low-risk PCs was significantly higher than that of the high-risk PCs. The AUC values for 1, 3, and 5 years were 0.774, 0.700, and 0.847 in the training cohort **(Figure 3G)**, 0.724, 0.775, and 0.840 in the internal validation cohort **(Figure 3H)**, and 0.729, 0.719, and 0.822 in the entire TCGA cohort** (Figure 3I)**, respectively. These results indicated that the model has good predictive performance. Risk score curves and survival status scatter plots showed that patients in the high-risk group had lower survival rates than those in the low-risk group from the training cohort **(Figure 3J)**, internal validation cohort **(Figure 3K)**, and the entire TCGA cohort **(Figure 3L)**.

**Figure 4:**
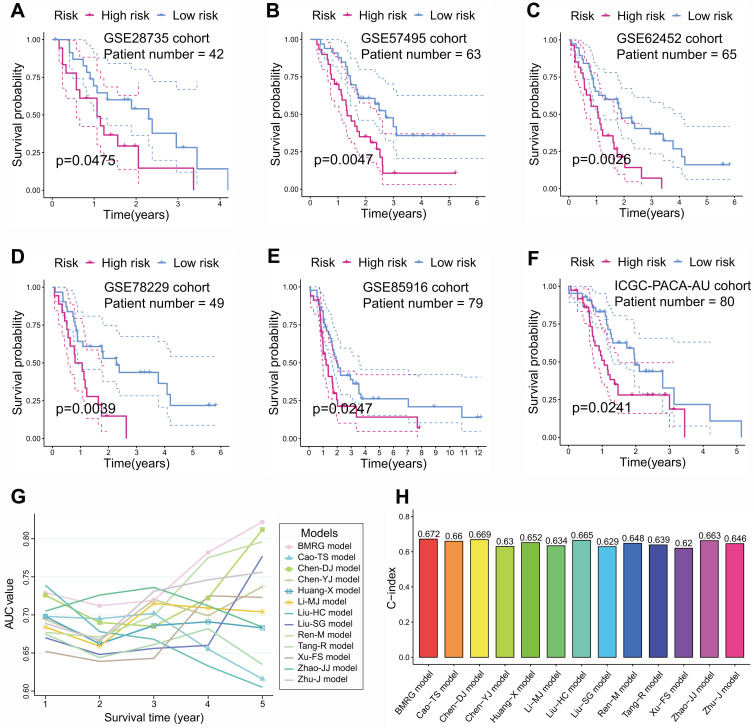
External validation and comparison of basement membrane-related prognostic model. In external validation cohorts GSE28735 **(A)**, GSE57495 **(B)**, GSE62452 **(C)**, GSE78229 **(D)**, GSE85916 **(E)**, and ICGC-PACA-AU **(F)**, Kaplan-Meier curves showed that the survival time of low-risk pancreatic cancer (PC) was notably higher than that of high-risk PC. The AUC value **(G)** and C-index **(H)** of basement membrane-related prognostic model were higher than those of the other twelve published models.

To illustrate the reliability of the model, GSE28735 **(Figure 4A)**, GSE57495 **(Figure 4B)**, GSE62452 **(Figure 4C)**, GSE78229 **(Figure 4D)**, GSE85916 **(Figure 4E)**, and ICGC-PACA-AU **(Figure 4F)** datasets, serving as external validation cohorts, showed that PCs with high-risk score had significantly lower survival times than those with low-risk score. In addition, by comparing the AUC value and C-index with published prognostic models for PC^33^-^44^, we found that BM-related prognostic model has better predictive performance **(Figures 4G and H)**.

### 3.3 Clinicopathological characteristics correlation, independent prognostic factors analysis, and nomogram prediction model construction

**Figure 5:**
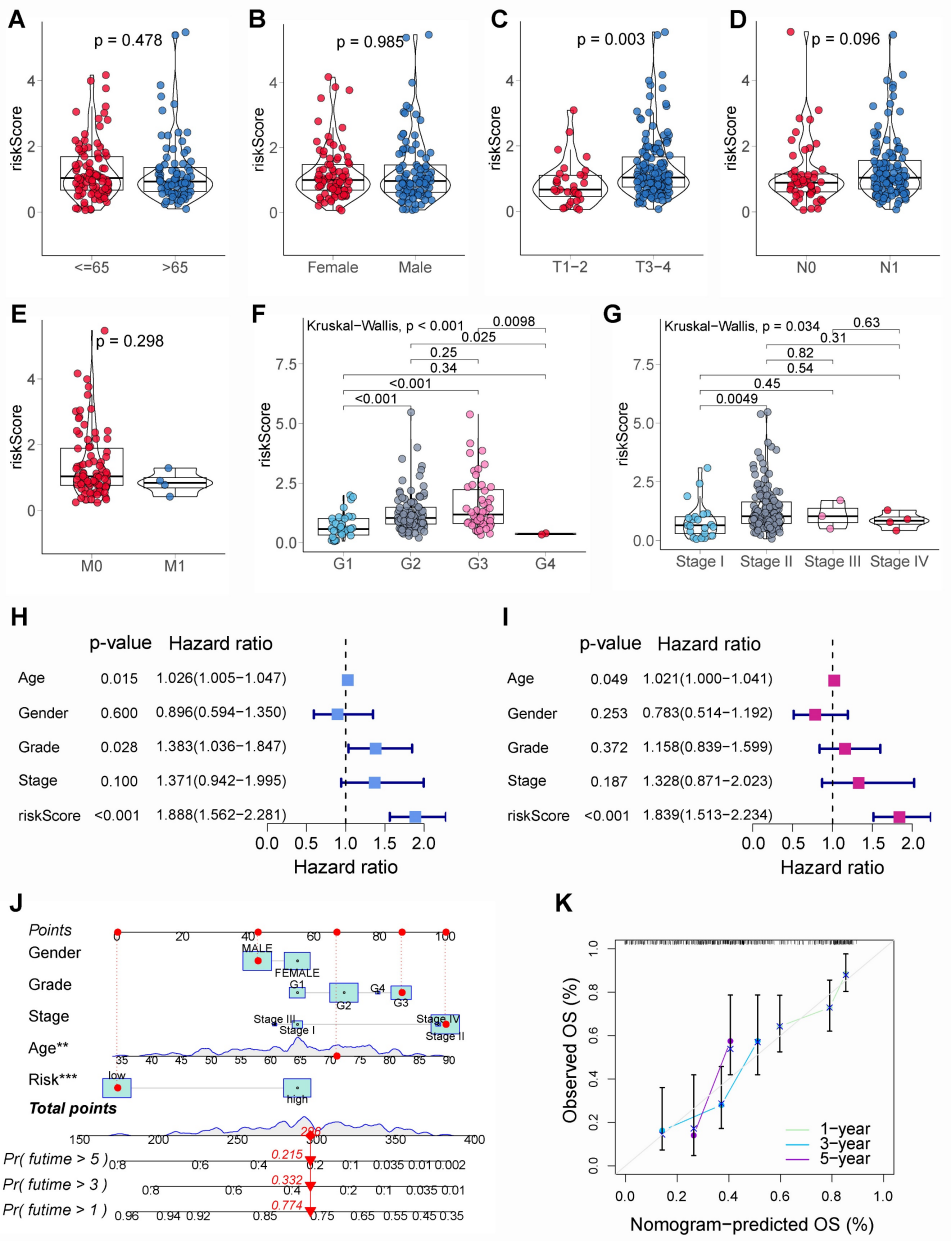
Clinicopathological characteristics correlation, independent prognostic analysis, and nomogram prediction model construction. The difference of risk score in various age groups **(A)**, gender groups **(B)**, T stages **(C)**, N stages **(D)**, M stages **(E)**, pathological grades **(F)**, and clinical stages **(G)**. **(H)** The univariable Cox regression showed that age, pathological grade, and basement membrane (BM)-related risk score were significantly associated with the prognosis of pancreatic cancer (PC). **(I)** The multivariable Cox regression showed that age and BM-related risk score were the independent prognostic factors of PC. **(J)** Nomogram prediction model was built using clinicopathological characteristics and BM-related risk score. **(K)** The calibration curve showed that the 1, 3, and 5-year survival rates predicted by the nomogram model were relatively close to the true survival rates.

The relationship between BM-related risk scores and clinicopathological characteristics was further assessed. Results showed that there were no significant differences in risk scores in the different ages **(Figure 5A)**, genders **(Figure 5B)**, N stages **(Figure 5D)**, and M stages **(Figure 5E)**. Nevertheless, in patients with higher T stages **(Figure 5C)**, pathological grades **(Figure 5F)**, and clinical stages **(Figure 5G)**, the risk score was notably higher. The univariable and multivariable Cox regression suggested that age and risk score were independent adverse prognostic factors for PCs **(Figures 5H and I)**. Then, a nomogram model was constructed using risk score and clinicopathological characteristics to better predict PCs' prognosis **(Figure 5J)**. The calibration curves showed that the 1, 3, and 5-year survival rates projected by the nomogram were comparatively near to the true survival rates **(Figure 5K),** suggesting that nomogram model had an excellent prediction performance.

### 3.4 GSEA

**Figure 6:**
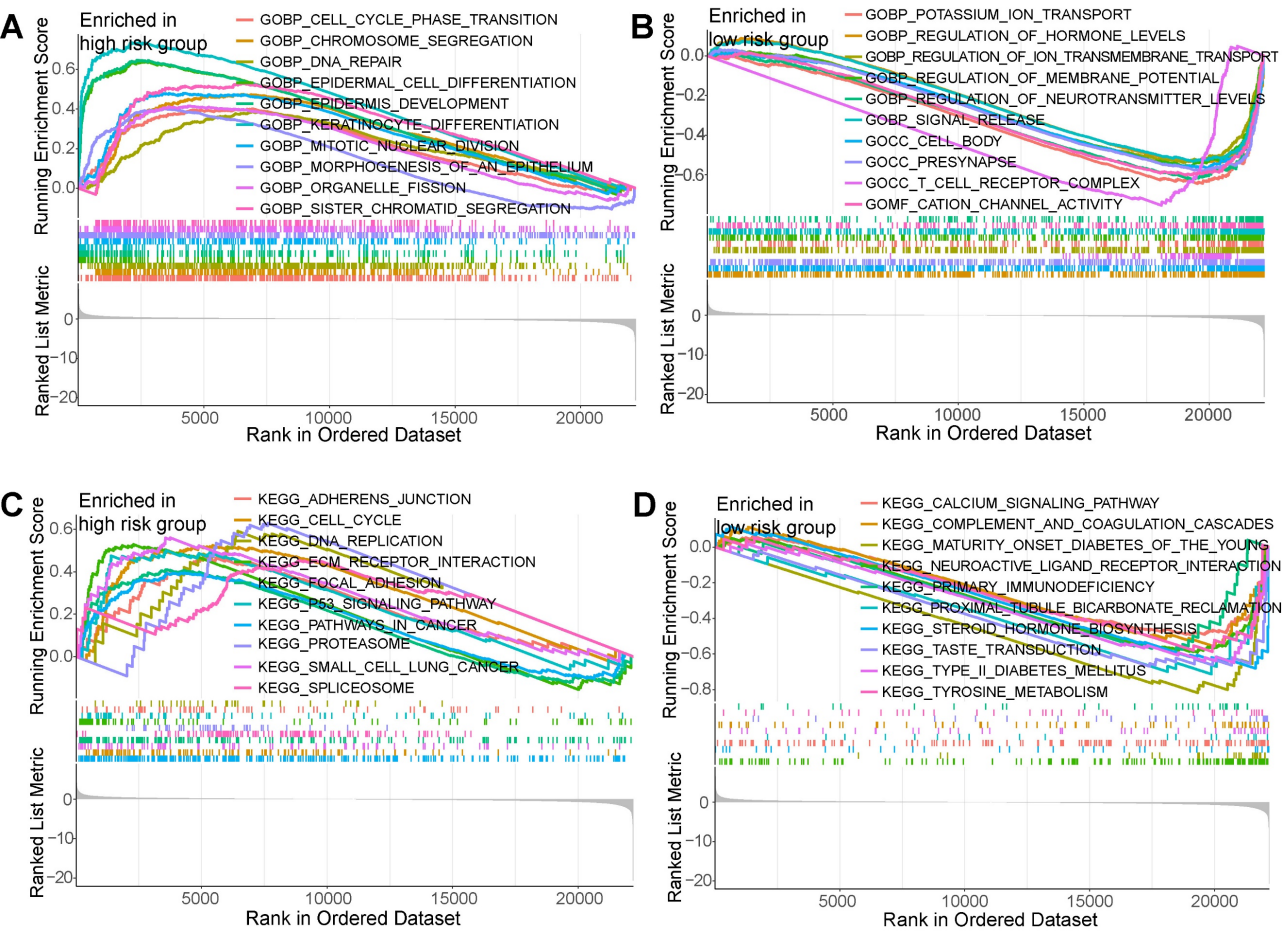
Gene set enrichment analysis (GSEA). GSEA showed the top 10 significantly enriched pathways of the high-risk group **(A)** and the low-risk group **(B)** based on the gene set “c5.go.v7.5.1.symbols.gmt”. GSEA showed the top 10 significantly enriched pathways of the high-risk group **(C)** and the low-risk group **(D)** based on the gene set “c2.cp.kegg.v7.5.1.symbols.gmt”.

To seek the potential diversities within biological behavior between different risk subgroups, GSEA was employed. Results showed that based on “c5.go.v7.5.1.symbols.gmt” gene set, the enrichment pathways within high-risk PCs encompassed “epidermis development”, “keratinocyte differentiation”, “mitotic nuclear division”, “cell substrate junction”, “chromosomal region”, and “DNA packaging complex” **(Figure 6A)**; the enrichment pathways within low-risk PCs encompassed “regulation of hormone levels”, “regulation of ion transmembrane transport”, “regulation of membrane potential”, “cation channel activity”, and “T cell receptor complex” **(Figure 6B)**. Based on “c2.cp.kegg.v7.5.1.symbols.gmt” gene set, the enrichment pathways within high-risk PCs encompassed “ECM receptor interaction”, “focal adhesion”, “P53 signaling pathway”, “pathways in cancer”, and “small cell lunger cancer” **(Figure 6C)**; the enrichment pathways within low-risk PCs encompassed “drug metabolism cytochrome P450”, “maturity onset diabetes of the young”, “neuroactive ligand receptor interaction”, and “proximal tubule bicarbonate reclamation” **(Figure 6D)**. It could be found that the enrichment pathways of the high-risk subgroup are similar with the biological processes involved in differentially expressed BMRGs, while the enrichment pathways in the low-risk subgroup is mainly related to normal physiological function and immunity.

### 3.5 Tumor microenvironment and immunotherapy

**Figure 7:**
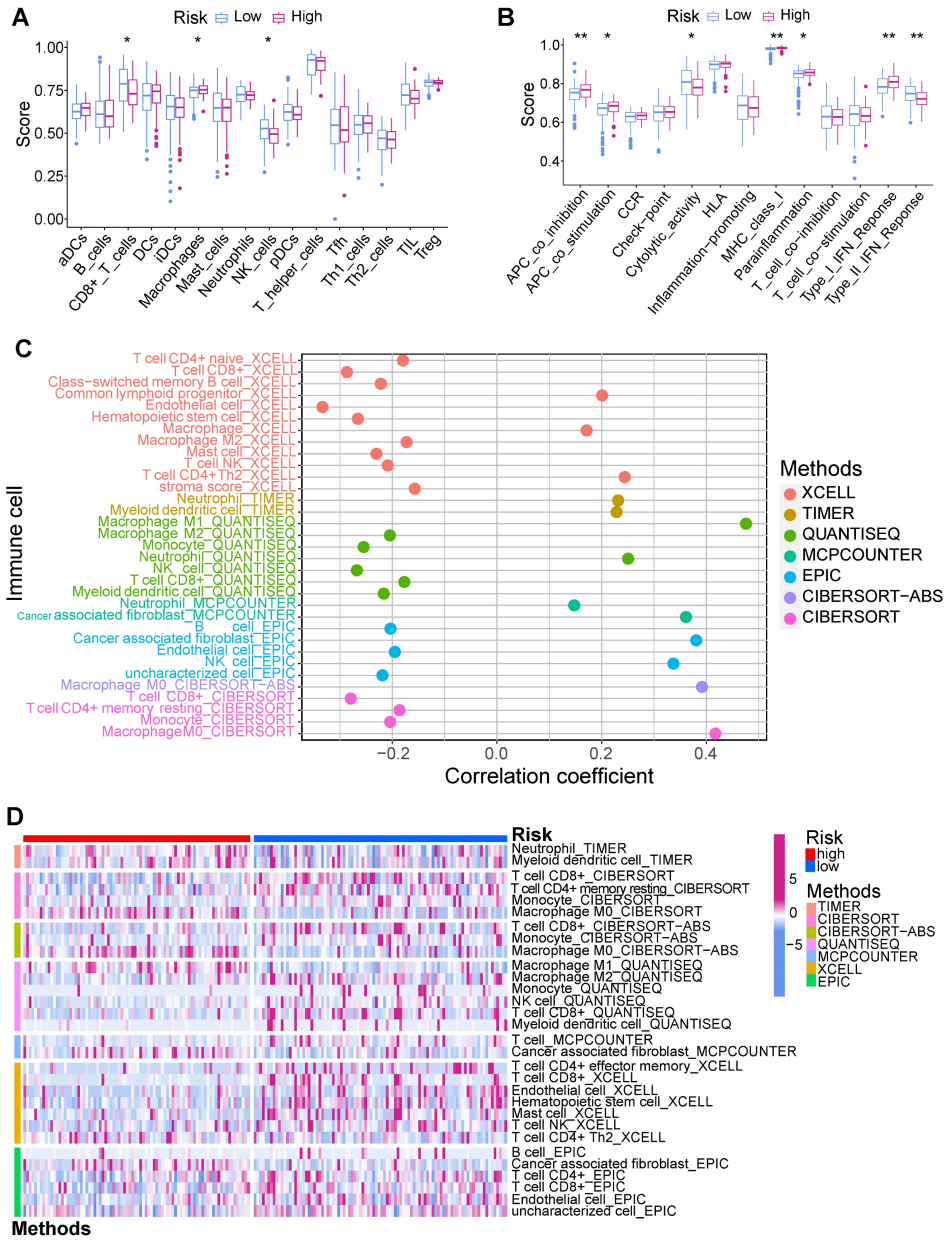
Immune infiltration analysis. **(A)** Single-sample gene set enrichment analysis (ssGSEA) showed that CD8+ T cells and NK cells had higher infiltration levels in the low-risk group, while macrophages had significantly higher infiltration levels in the high-risk group. **(B)** ssGSEA showed that Type II IFN response in the low-risk group had a significantly higher score, while APC co-inhibition, APC co-stimulation, MHC class I, parainflammation, and Type I IFN response in the high-risk group had significantly higher scores. XCELL, TIMER, QUANTISEQ, MCPCOUNTER, EPIC, CIBERSORT-ABS, and CIBERSORT algorithms were employed to assess the correlation of BM-related risk score and immune cell subpopulations **(C)**, as well as the difference in the infiltration levels of immune cell subpopulations between high- and low-risk subgroups **(D)**.

Tumor immune microenvironment was evaluated using multiple algorithms in this study. The ssGSEA manifested that the CD8+ T cells, NK cells, and Type II IFN response within low-risk PCs exhibited a notably elevated level **(Figure 7A)**, while macrophages, APC co-inhibition, APC co-stimulation, MHC class I, and parainflammation within high-risk PCs exhibited a notably elevated level **(Figure 7B)**. Based on the infiltrated scores of immune cell subpopulations obtained via TIMER database, the correlation of BM-related risk score and immune cell subpopulations were further assessed **(Figures 7C and D)**. Results demonstrated a significantly negative correlation between BM-related risk score and CD8+ T cells, NK cells, B cells, monocytes, and endothelial cells, and these cell subpopulations had notably higher abundance in low-risk PCs. However, macrophage M0, neutrophils, and CAFs showed a substantially positive connection with BM-related risk score, and these cell subpopulations had substantially higher levels in high-risk PCs.

**Figure 8:**
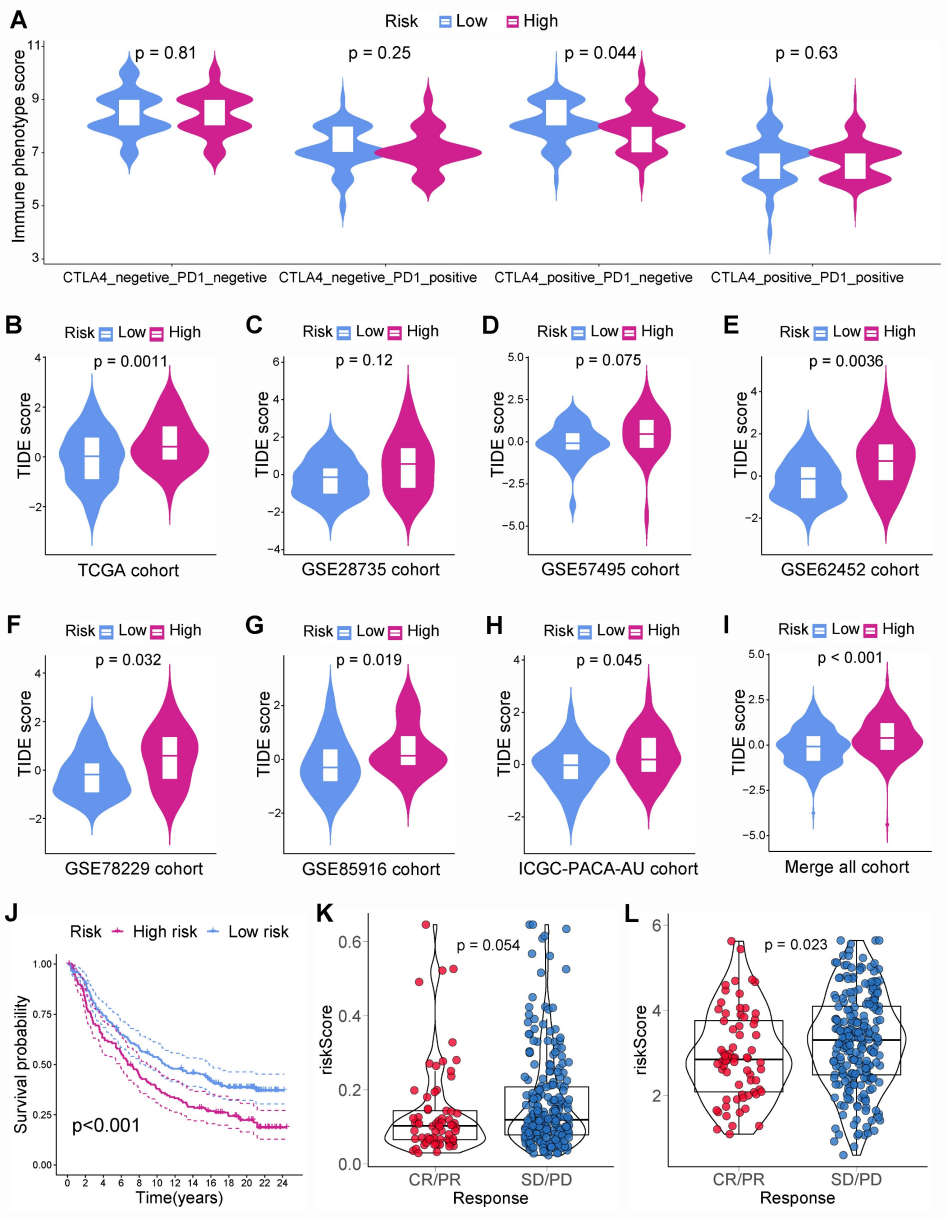
Immunotherapy response. **(A)** The immune phenotype score (IPS) for CTLA-4 in the low-risk PC was notably higher than that in high-risk PC. PC from TCGA **(B)**, GSE28735 **(C)**, GSE57495 **(D)**, GSE62452 **(E)**, GSE78229 **(F)**, GSE85916 **(G)**, ICGC-PACA-AU **(H)**, and merged all cohort **(I)** showed that tumor immune dysfunction and exclusion (TIDE) score was notably higher in high-risk group. **(J)** The basement membrane-related prognostic model was applied to Imvigor210 cohort, the survival time in low-risk group was notably higher than that in high-risk group. **(K)** The risk score of patients in the immunotherapy responder group was lower than that of the non-responder group, and the difference is very close to statistical significance. **(L)** PLAU expression in the immunotherapy response group was significantly lower than that in the non-response group.

Besides, immunotherapy responses in different subgroups were investigated. The IPS for CTLA-4 within low-risk PCs was noticeably greater than that within high-risk PCs, suggesting that immunotherapy could be more beneficial for low-risk PCs, especially CTLA-4 blockers **(Figure 8A)**. To validate this finding, TIDE scores were computed in various subgroups. PCs from TCGA cohort suggested that low-risk PCs had noticeably lower TIDE scores, which indicated that low-risk PCs were more sensitive to immunotherapy **(Figure 8B)**. Similarly, the external cohorts also suggested that low-risk PCs had lower TIDE scores **(Figures 8C-I)**. Besides, the immunotherapy dataset Imvigor210 including 348 UCs was employed further to evaluate the connection between BM-related risk score and immunotherapy. The prognosis of UCs with a low BM-related score was better than that of UCs with a high BM-related score, indicating that the BM-related prognostic model may apply to different tumors **(Figure 8J)**. The BM-related scores within the immunotherapy responder group were smaller than those within the non-responder group **(Figure 8K)**. Further analysis showed that PLAU within immunotherapy response group had a considerably lower expression than that within immunotherapy non-response group **(Figure 8L)**. The above results indicated that PCs with a low BM-related score had higher infiltration of CD8+ T cells and were more likely to benefit from immunotherapy.

### 3.6 Chemotherapy and targeted therapy sensitivity

**Figure 9:**
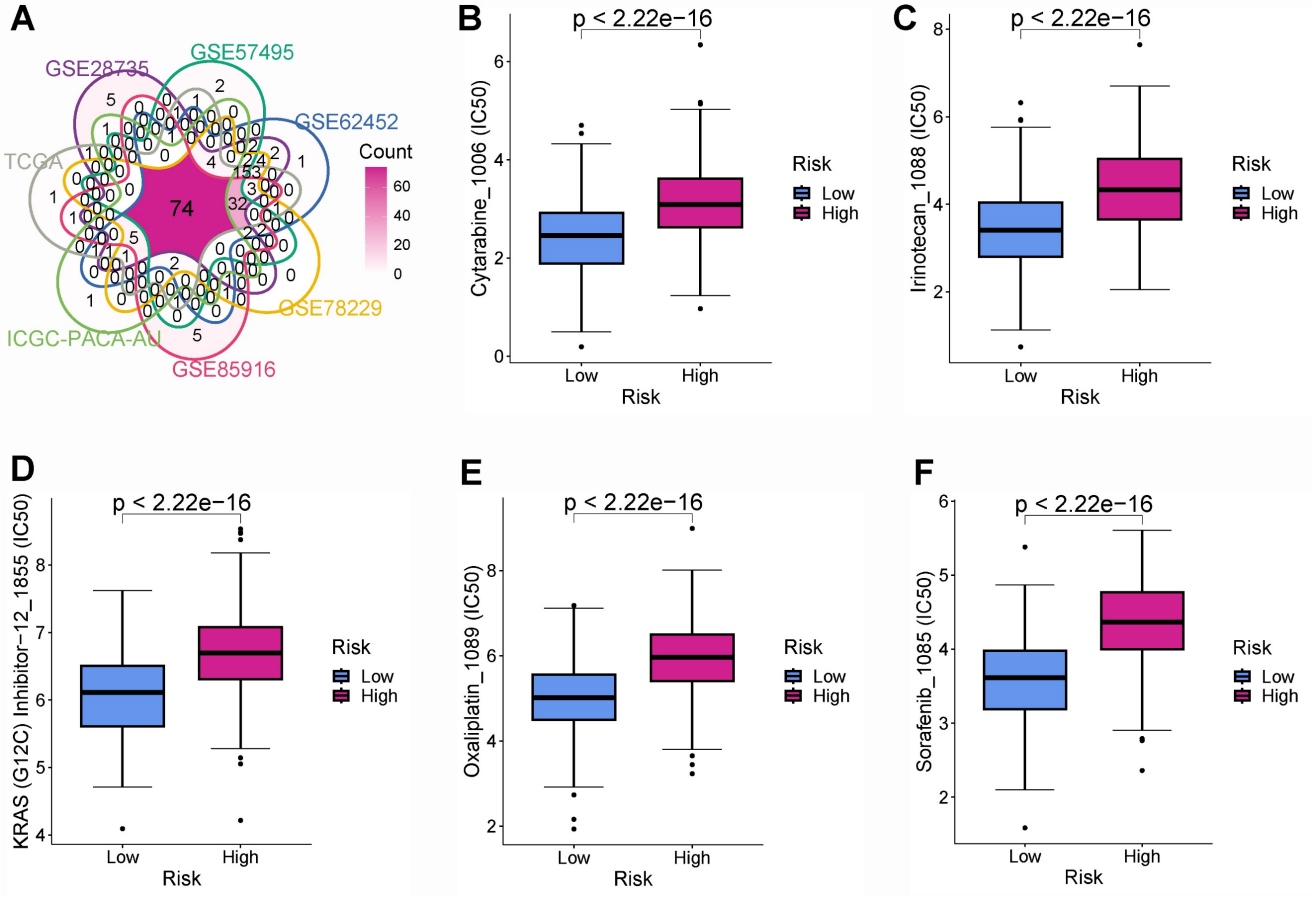
Drug sensitivity analysis. **(A)** Intersection of drug molecules with significantly different sensitivities between high- and low-risk subgroups from all cohorts. The low-risk group was more sensitive to cytarabine **(B)**, irinotecan **(C)**, KRAS(G12C) Inhibitor-12 **(D)**, oxaliplatin **(E)**, and sorafenib **(F)**.

Chemotherapy and targeted therapies are important in improving PC prognosis. Nevertheless, primary or secondary drug tolerance can cause individual differences in efficacy. Therefore, identifying the highly sensitive drugs for each PC patient is essential to improve the effectiveness of drug therapy and develop a personalized treatment plan. This study identified 149, 160, 141, 160, 152, 130, and 90 drug molecules with significantly different sensitivity in various risk group from the TCGA, GSE28735, GSE57495, GSE62452, GSE78229, GSE85916, and ICGC-PACA-AU cohorts, respectively **(Table S5)**. After the intersection of all the cohorts, there were 74 drug molecules with significant sensitivity differences **(Figure 9A)**. PCs with a lower BM-related score were more sensitive to cytarabine **(Figure 9B)**, irinotecan **(Figure 9C)**, KRAS(G12C) Inhibitor-12 **(Figure 9D)**, oxaliplatin **(Figure 9E)**, and sorafenib **(Figure 9F)**.

### 3.7 Expressed and prognostic validation by clinical cohort

**Figure 10:**
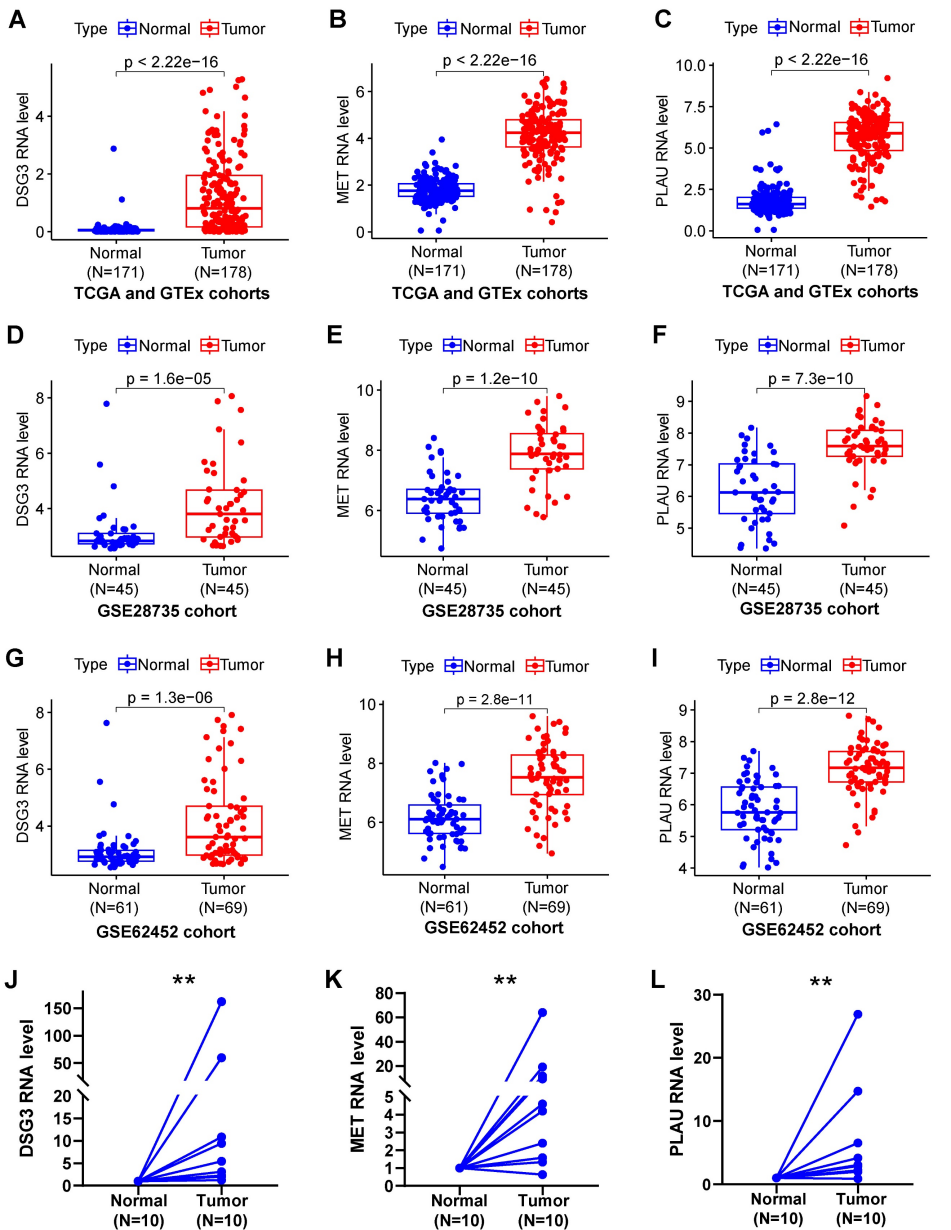
DSG3, MET, and PLAU expression. Pancreatic cancer from TCGA and GTEx **(A-C)**, GSE28735 **(D-F)**, and GSE62452 **(G-I)** datasets suggested that DSG3, MET, and PLAU were notably up-regulated in PC tissues in comparison to normal tissues. PCR showed that DSG3 **(J)**, MET **(K)**, and PLAU **(L)** had significantly higher RNA levels within PC tissues than normal tissues. (ns P-value > 0.05; * P-value < 0.05; ** P-value < 0.01; *** P-value < 0.001).

Samples from TCGA and GTEx **(Figures 10A-C)**, GSE28735 **(Figures 10D-F)**, and GSE62452 **(Figures 10G-I)** datasets suggested that DSG3, MET, and PLAU were notably up-regulated in PC tissues in comparison to normal tissues. To further verify the expression of DSG3, MET, and PLAU. we obtained 10 pairs of PC and normal tissues from the First Affiliated Hospital of Dalian Medical University and further performed PCR experiments. Results showed that DSG3, MET, and PLAU had significantly higher RNA levels within PC tissues than normal tissues **(Figures 10J-L)**.

**Figure 11:**
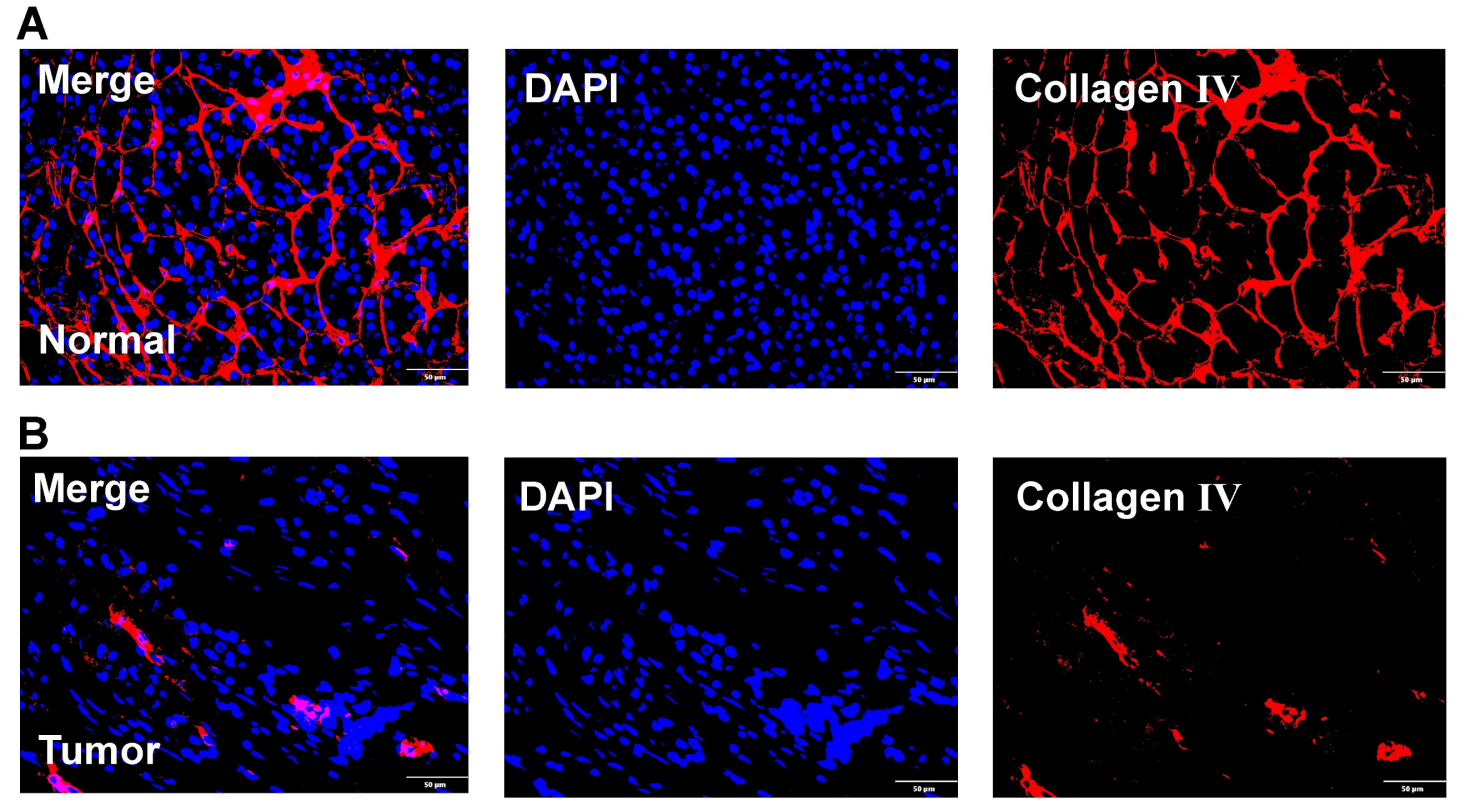
Immunofluorescence. **(A)** Immunofluorescence image of basement membrane (BM) structure in normal tissues with Collagen IV (red) and nuclei (DAPI; blue). **(B)** Immunofluorescence image of BM structure in pancreatic cancer tissues with Collagen IV (red) and nuclei (DAPI; blue).

**Figure 12:**
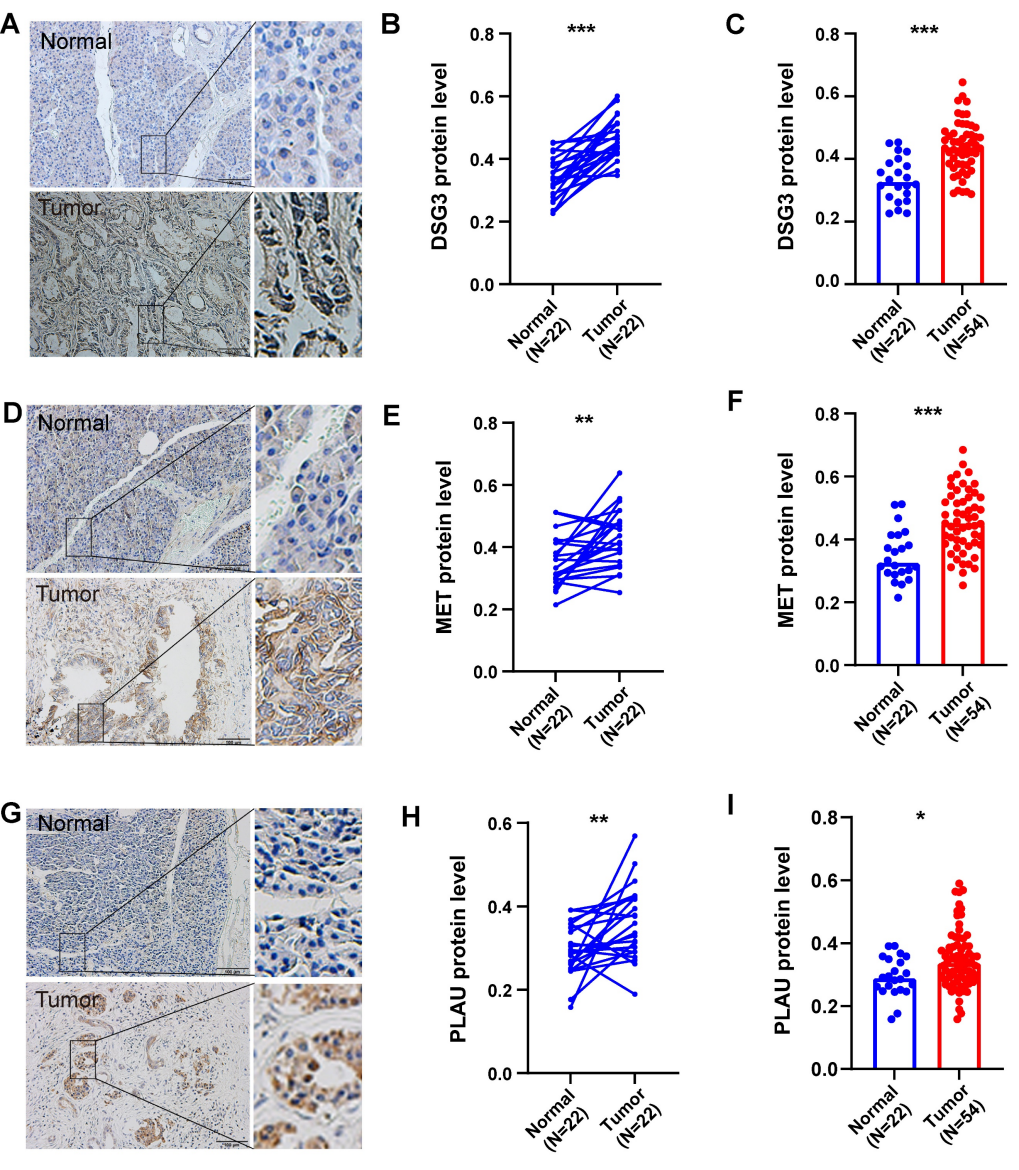
Immunohistochemistry. Immunohistochemical images of DSG3 **(A)**, MET **(D)**, and PLAU **(G)** in pancreatic cancer (PC) and normal tissues. The immunohistochemistry results of 24 pairs of PC and normal tissues indicated that protein levels of DSG3 **(B)**, MET **(E)**, and PLAU **(H)** in PC tissues were notably higher than those in normal tissues. The immunohistochemistry results of 54 PC and 24 normal tissues indicated that protein levels of DSG3 **(C)**, MET **(F)**, and PLAU **(I)** in PC tissues were notably higher than those in normal tissues. (ns P-value > 0.05; * P-value < 0.05; ** P-value < 0.01; *** P-value < 0.001).

**Figure 13:**
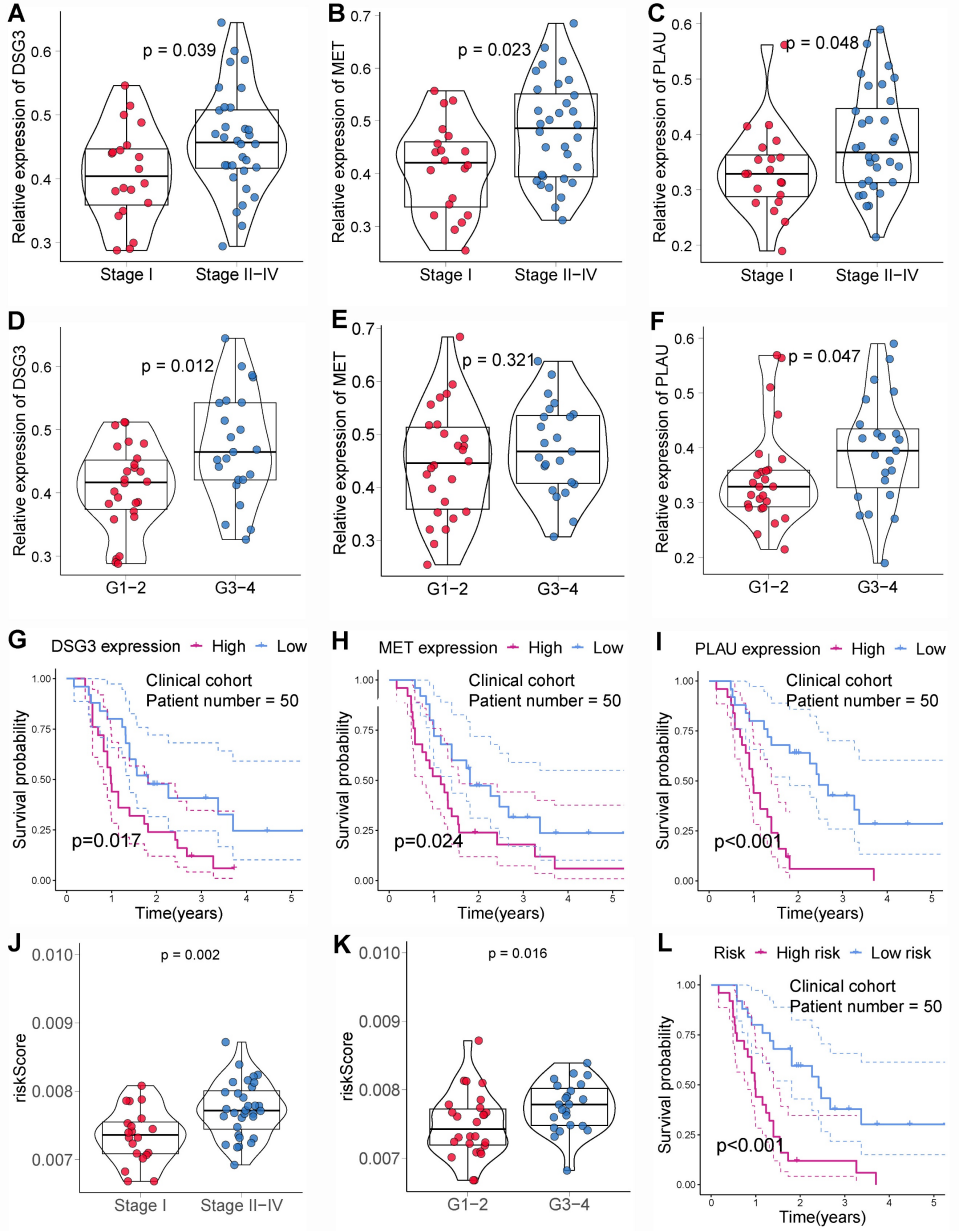
Clinical cohort validation of basement membrane-related prognostic model. The expression levels of DSG3 **(A)**, MET **(B)**, and PLAU **(C)** between stage I and stage II-IV pancreatic cancer (PC). The expression levels of DSG3 **(D)**, MET **(E)**, and PLAU **(F)** between pathological grade 1-2 and pathological grade 3-4 PC. Kaplan-Meier curves showed that the prognoses of DSG3 **(G)**, MET **(H)**, and PLAU **(I)** high-expression subgroup were notably worse than those of the low-expression subgroup. **(J)** The BM-related risk score of PCs with stage II-IV was notably higher than that of PCs with stage I. **(K)** The BM-related risk score of PCs with pathological grade 3-4 was notably higher than that of PCs with pathological grade 1-2. **(L)** Kaplan-Meier curves showed that PCs with a high BM-related risk score had a notably worse prognosis than PCs with a low BM-related risk score.

In addition, we also obtained paraffin-embedded pathological sections of 54 PC tissues and 22 normal tissues from the First Affiliated Hospital of Dalian Medical University. Meanwhile, clinicopathological and survival information of 54 patients with PC was collected. To investigate the differences in BM structure between PC and normal tissues, we performed immunofluorescence staining for the classical BM marker type IV collagen^23^,^57^. Results showed that BM structure was intact and regular in normal tissues, whereas it was fragmented and irregular in PC tissues (**Figures 11A-B**). Immunohistochemistry was performed to investigate to the expression of model genes. And results showed that DSG3, MET, and PLAU had higher protein levels within PC tissues in comparison to adjacent tissues **(Figures 12A-I)**. Clinical survival information and DSG3, MET, and PLAU expression of 54 PC samples were further combined. Results showed that DSG3, MET, and PLAU had significantly higher levels within advanced PCs **(Figures 13A-C)**. DSG3 and PLAU were had significantly higher levels within higher pathological grades **(Figures 13D and F)**. MET had a higher level in PC with higher pathological grade, but no statistical difference, which could be related to the limited patient number **(Figure 13E)**. KM curves suggested that up-regulation of DSG3, MET, and PLAU was linked to a poorer outcome **(Figures 13G-I)**. Subsequently, this study further calculated the BM-related risk score for each PC patient based on the expression of DSG3, MET, and PLAU. The BM-related risk score was remarkably higher within higher clinical stage and pathological grade **(Figures 13J and K)**. KM curves indicated that PCs with a high BM-related risk score had a poorer outcome **(Figure 13L)**.

### 3.8 DSG3 knockdown inhibited the proliferation, migration, and invasion of PC cells

**Figure 14:**
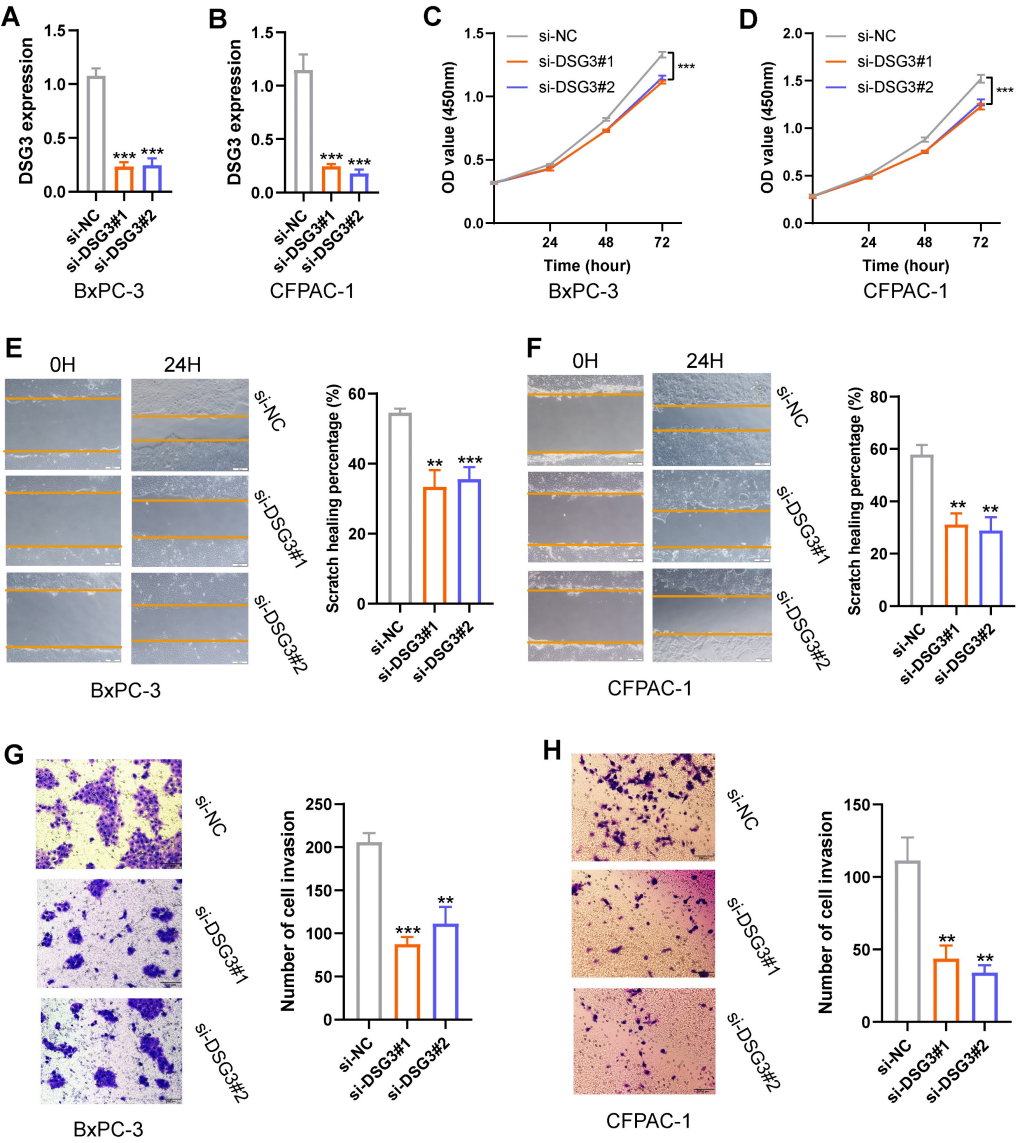
*In vitro* experiment. PCR showed that DSG3 was successfully knocked down in BxPC-3 **(A)** and CFPAC-1 **(B)** cells. CCK-8 assays indicated that DSG3 knockdown could significantly restrain the proliferation of BxPC-3 **(C)** and CFPAC-1 **(D)** cells. Scratch assays indicated that the migration ability of BxPC-3 **(E)** and CFPAC-1 cells **(F)** was significantly inhibited after DSG3 knockdown. Transwell invasion assays indicated that the invasion ability of BxPC-3 **(G)** and CFPAC-1 cells **(H)** was significantly inhibited after DSG3 knockdown. (ns P-value > 0.05; * P-value < 0.05; ** P-value < 0.01; *** P-value < 0.001).

Based on the above findings, DSG3, MET, and PLAU are upregulated in PC and are associated with poor prognosis. MET and PLAU have been extensively reported in various cancers, including PC^58^-^61^. Cancers with poorer prognoses typically exhibit enhanced proliferation, migration, and invasion capabilities in their cells. Therefore, this study further explored the function of DSG3 in PC cells. The siRNA targeting DSG3 was transfected into PC cells BxPC-3 and CFPAC-1. PCR results showed that DSG3 was successfully knocked down in BxPC-3 and CFPAC-1 cells** (Figures 14A and B)**. CCK-8 assays indicated that DSG3 knockdown could suppress the proliferation of BxPC-3 and CFPAC-1 cells **(Figures 14C and D)**. Scratch assays demonstrated that DSG3 knockdown could significantly reduce the migration of BxPC-3 and CFPAC-1 cells **(Figures 14E and F)**. Transwell invasion assays showed that DSG3 knockdown could suppress the invasion of BxPC-3 and CFPAC-1 cells** (Figures 14G and H)**. These results suggested that DSG3 knockdown may inhibit the proliferation, migration, and invasion of PC cells.

### 3.9 Potential therapeutic drug and molecular docking

**Figure 15:**
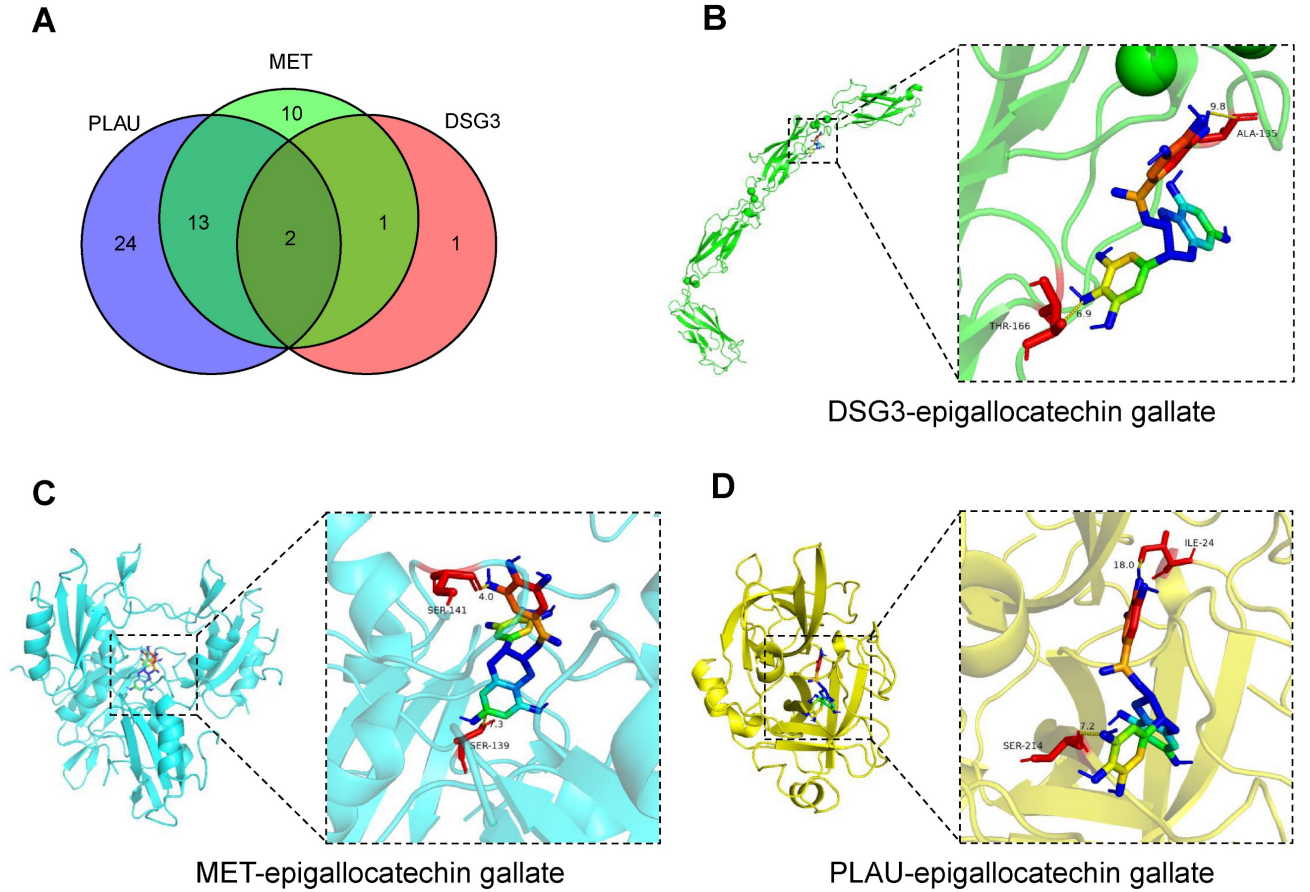
Candidate drug prediction and molecular docking. **(A)** The intersection of drug molecules targeting DSG3, MET, and PLAU. Molecular docking results of epigallocatechin gallate with DSG3 **(B)**, MET **(C)**, and PLAU **(D)**.

The comparative toxicogenomics database was used to search for drug molecules that could target DSG3, MET, and PLAU in pancreatic tumors, obtaining 4, 26, and 39 drug molecules, respectively **(Table S6)**. After intersection, two drug molecules epigallocatechin gallate and tobacco smoke pollution were obtained **(Figure 15A)**. Based on relevant data from the comparative toxicogenomics database, epigallocatechin gallate can decrease the expression levels of DSG3, MET, and PLAU. Epigallocatechin gallate is an active compound in green tea, which has been shown to inhibit cancer cell proliferation and angiogenesis, and is a potential therapeutic agent for malignant tumors^62^. Subsequently, we performed molecular docking of epigallocatechin gallate with DSG3, MET, and PLAU **(Figures 15B-D)**. The binding energy of epigallocatechin gallate with DSG3, MET, and PLAU were -8.0 kcal/mol, -10.6 kcal/mol, and -9.5 kcal/mol, respectively **(Table S7)**, indicating that epigallocatechin gallate has a strong binding activity with DSG3, MET, and PLAU and may be used as a potential therapeutic agent for PC.

## 4. Discussion

PC is characterized by substantial malignancy, strong invasiveness, and extremely poor prognosis^63^. Additionally, PC has an insidious onset and atypical early symptoms. About 80-85% of PCs miss out on the chance of undergoing surgery when diagnosed^6^. Despite receiving surgical treatment and adjuvant treatment after surgery, about 70% of PCs will still have recurrence and metastasis within 2 years after surgery^64^-^66^. Invasiveness and metastases are important features of malignancies and are responsible for 66%-90% of deaths in cancer patients^67^. BM is formed by cross-linking macromolecules in the ECM and acts as a barrier to cancer cell aggressiveness and metastases. The destruction and structural disorder of BM are important processes for PC cells to invade the surrounding stroma^57^.

We found 46 differential BMRGs between PC and normal tissues, which were notably enriched in “basement membrane”, “focal adhesion”, “ECM-receptor interaction”, and “PI3K-Akt signaling pathway”. Focal adhesion can attach the ECM to the cytoskeleton inside and is crucial for preserving cell survival, proliferation, differentiation, and movement by regulating cell morphology and intracellular signal transduction^68^,^69^. Studies manifested that the dysregulation of focal adhesion and ECM-receptor interaction is closely associated with tumor cell shedding, adhesion, invasion, and metastases^70^,^71^. As one of the important signaling within cells, PI3K/Akt could enhance cell viability, prevent apoptosis, etc.^72^,^73^. Besides, PI3K/Akt signaling is crucial in the pathological process of human cancer. Its abnormal activation could cause the change of a series of downstream proteins, thereby promoting cancer cell growth, invasiveness, and metastases^74^-^76^ and inducing the formation of tumor neovascularization^77^,^78^. Therefore, BMRGs may participate in the above processes to facilitate the occurrence and progression of cancer.

To stratify PCs and assess prognosis, the BM-related risk score model (including MET, DSG3, and PLAU) was constructed and validated using different public databases and clinical cohort. MET is a proto-oncogene encoding the mesenchymal-to-epithelial transition protein which belongs to the tyrosine kinase receptor. As a ligand, hepatocyte growth factor may attach with the extracellular region of MET protein, then activate the kinase and phosphorylate the tyrosine. Phosphorylated MET protein can recruit a variety of effector molecules and activate a series of downstream signaling, including PI3K/Akt and ERK/MAPK^58^,^79^,^80^. The high expression of MET was found within various malignancies, encompassing head and neck squamous cell carcinoma^81^,^82^, lung cancer^83^,^84^, esophageal cancer^85^, gastric cancer^80^,^86^,^87^, colorectal cancer^88^,^89^, hepatocellular carcinoma^90^, PC^91^, and kidney cancer^92^,^93^. This study showed that MET in PC tissues was significantly up-regulated and linked to a poor outcome. DSG3, as one kind of calcium-bound transmembrane glycoprotein among members of the cadherin superfamily, plays an intercellular connection role in desmosomes, and participates in many signaling pathways^94^. Xin *et al.*^95^ showed that DSG3 was down-regulated within oral squamous cell carcinoma, and low DSG3 expression was linked to higher pathological grade and lymph node metastasis rate. However, some studies found that DSG3 had a cancer-promoting effect, and upregulation of DSG3 was related to tumor development and bad outcomes, such as head and neck cancer^96^ and esophageal cancer^97^. Therefore, DSG3's function in different tumors remains controversial. This study showed that DSG3 was substantially up-regulated within PC and related to higher TNM stage and worse outcomes. *In vitro* experiments showed that DSG3 knockdown could inhibit the proliferation, migration, and invasion of PC cells. PLAU encodes the production of urokinase-type plasminogen activator which belongs to one kind of serine protease and could promote the conversion of plasminogen to plasmin^98^,^99^. Studies showed that PLAU may directly or indirectly degrade the ECM elements, including laminins and collagen fibers, by activating matrix metalloproteinases, thereby facilitating tumor cell metastasis and angiogenesis^100^,^101^. A meta-analysis^102^ manifested that PLAU was involved in shorter overall and recurrence-free survival of gastroesophageal cancer. This study indicated that PLAU was notably up-regulated within PC and related to PC progression and poor prognosis.

Tumor immune environment is currently a hot topic in the field of oncology and is related to cancer progression and immunotherapy response. This study manifested that PCs with a low BM-related score had higher infiltrations of CD8+ T cells, NK cells, and B cells, while PCs with a high BM-related score had higher infiltrations of neutrophils, and CAFs. As one of the main effector cells of anti-cancer immunity, CD8+ T cells could destroy tumor cells through cytotoxicity and inhibit tumor angiogenesis by secreting interferon gamma^103^. High abundance of CD8+ T cells is generally linked to higher immunotherapy response and better outcome for cancer patients, such as colon cancer^104^, gastric cancer^105^, and PC^106^. This was consistent with our findings that PCs with high levels of CD8+ T cells had a better prognosis and a greater likelihood of benefit from immunotherapy. NK cells are involved in tumor immune surveillance and can quickly recognize cancer cells without prior sensitization. It could directly kill cancer cells through releasing cytotoxic granules comprising perforin and granzyme, and could also destroy cancer cells through excreting immunomodulatory cytokines like nitric oxide and expressing other tumor necrosis factor family members^107^-^109^. In contrast to T cells, there is debate concerning B cells' involvement in cancer^110^. Zhang *et al.*^111^ manifested that the high abundance of B cells within lung adenocarcinoma tissue was connected with a good outcome. Iglesia *et al.*^112^ investigated 11 tumors derived from TCGA platform and revealed that the elevated B cell levels predicted high survival times in most tumors. However, there are also studies showing that B cells can promote tumor development. Yang *et al.*^113^ suggested that B cells with STAT3 activation could promote tumor angiogenesis and thereby promote tumor development, and therefore can be used as a potential treatment target. Woo *et al.*^114^ found that the abundance of CD20+ B cells within prostate cancer tissue was increased compared with normal prostate tissue and was linked to prostate cancer progression and recrudesce. Chen *et al.*^115^ identified two main B cell subtypes: naive-like B cells and plasma-like B cells, utilizing single-cell RNA sequencing. Naïve-like B cells have inhibitory effects on the proliferation of lung cancer cells, while plasma-like B cells can suppress lung cancer cell growth in the early stages and promote lung cancer cell growth in the advanced stages. Therefore, the function of B cells on tumors may be related to their different subtypes. Neutrophils, as first responders to infection and inflammation, are an essential part for innate immunity, and neutrophils' impact on tumors has currently attracted increasing attention from oncologists^116^. Many studies showed that neutrophils have pro-cancer effects by promoting tumor growth and metastasis^117^-^119^, angiogenesis^120^,^121^, remodeling ECM^122^, and suppressing anti-tumor immunity^123^. However, several studies manifested that neutrophils could restrain tumor cell proliferation and therefore have the potential for anti-tumor effects^124^,^125^. The contradictory roles displayed by neutrophils in tumor progression may be the result of their different plasticity and functional states^126^,^127^. In addition to immune cells, CAFs are also one an important cellular elements within TME and participate in a series of cancer-promoting processes, like cancer cell migration, chemotherapy and radiotherapy resistances, and immune suppression^128^-^131^. Glentis *et al.*^19^ found that CAFs can expand the gaps of BM by applying mechanical forces such as contraction and stretching, thereby assisting tumor cell metastasis.

The current first-line treatment for PC is the FOLFIRINOX regimen or gemcitabine plus albumin-paclitaxel. Frustratingly, even with first-line chemotherapy, merely 30% of PCs are sensitive^132^. Drug resistance, either primary or secondary, is the primary reason for therapy failure and plays a vital function in the high mortality rate of cancer patients. It is crucial to understand the potential molecular mechanism of drug resistance and to search for individualized sensitive drugs. Fridman *et al.*^133^ found that recombinant BM and laminin could promote lung cancer cell lines' tumorigenicity and chemotherapy resistance. This study demonstrated that BM-related risk score was linked to chemotherapy and targeted therapy sensitivity. PCs with a low BM-related score were more sensitive to oxaliplatin, irinotecan, cytarabine, and KRAS(G12C) inhibitor-12. Oxaliplatin and irinotecan are two drugs in the FOLFIRINOX regimen. This may partially explain why PCs with a low BM-related score had a higher survival. An important feature of the pathogenesis of PC is KRAS mutation, with a mutation frequency of about 90%. KRAS codon 12 mutations (71%) are the most common, including G12D (42%), G12V (32%), G12R (15 %), G12C (1.5%), G12A (0.4%), and G12S (0.1%)^134^-^136^. Therefore, targeting KRAS mutations in PC therapy has a theoretical basis and prospect. There are currently early clinical studies on KRAS G12C in the treatment of PC, which have shown good results^134^,^136^,^137^.

Immunotherapy has developed rapidly in recent years, bringing new hope for cancer treatment. Immunotherapy has been used to improve the prognosis of numerous malignancies, comprising non-small cell lung cancer^138^, hepatocellular carcinoma^139^, renal carcinoma^140^, melanoma^141^, and esophageal squamous cell carcinoma^142^. Nevertheless, due to the complicacy and heterogeneity of tumorigenesis, the overall response rate for immunotherapy is low, and only 10%-30% of patients can benefit from immunotherapy^143^. Therefore, identifying patient subpopulations that are sensitive to immunotherapy is beneficial to the development of precision oncology medicine. This study showed that immunotherapy was more beneficial for PCs with a low BM-related score. Therefore, there may be a potential link between BM and tumor immunotherapy response. We also look forward to more research to explore in the future.

## 5. Conclusion

This study developed and validated a BM-related risk score model (including DSG3, MET, and PLAU) in PC using multiple public and clinical cohorts, with a good prediction efficiency for the prognosis, tumor immune environment, and therapy response. DSG3, MET, and PLAU were notably up-regulated within PC tissues and linked to a poor prognosis. And DSG3 knockdown markedly inhibited the proliferation, migration, and invasion of PC cells. Epigallocatechin gallate had a strong binding activity with DSG3, MET, and PLAU and may be used as a potential therapeutic agent for PC. These results offered novel insights into PC stratification and drug intervention.

## Supplementary Material

Supplementary tables.
