# Supplementary material for: Identification of basement membrane-related prognostic model associated with the immune microenvironment and synthetic therapy response in pancreatic cancer: integrated bioinformatics analysis and clinical validation
Source: J Cancer. 2024 Oct 14;15(19):6273–98. doi: 10.7150/jca.100891 (PMC11540510; doi:10.7150/jca.100891)
Supplement: Supplementary file 1 — Supplementary tables. [file jcav15p6273s1.zip › Table S7.docx]

**Table S7. Binding energy of epigallocatechin gallate with DSG3, MET, and PLAU**

| **Binding energy of epigallocatechin gallate with DSG3, MET, and PLAU** | | | |
| --- | --- | --- | --- |
| Targets | DSG3 | MET | PLAU |
| Binding energy (kcal/mol) | -8.0 | -10.6 | -9.5 |
