# Supplementary material for: Identification of basement membrane-related prognostic model associated with the immune microenvironment and synthetic therapy response in pancreatic cancer: integrated bioinformatics analysis and clinical validation
Source: J Cancer. 2024 Oct 14;15(19):6273–98. doi: 10.7150/jca.100891 (PMC11540510; doi:10.7150/jca.100891)
Supplement: Supplementary file 1 — Supplementary tables. [file jcav15p6273s1.zip › Table S1.docx]

**Table S1.** **Clinical information for patients with pancreatic cancer from TCGA dataset.**

| Characteristics |  | Number | Percentage (%) |
| --- | --- | --- | --- |
| Age (years) | <=65 | 96 | 51.9 |
|  | >65 | 89 | 48.1 |
| Gender | Male | 102 | 55.1 |
|  | Female | 83 | 44.9 |
| Clinical stage | Stage I | 21 | 11.5 |
|  | Stage II | 152 | 83.5 |
|  | Stage III | 4 | 2.2 |
|  | Stage IV | 5 | 2.7 |
| Pathological grade | G1 | 32 | 17.6 |
|  | G2 | 97 | 53.3 |
|  | G3 | 51 | 28.0 |
|  | G4 | 2 | 1.1 |
| Vital status | Survival | 85 | 45.9 |
|  | Death | 100 | 54.1 |
