# Supplementary material for: Identification of basement membrane-related prognostic model associated with the immune microenvironment and synthetic therapy response in pancreatic cancer: integrated bioinformatics analysis and clinical validation
Source: J Cancer. 2024 Oct 14;15(19):6273–98. doi: 10.7150/jca.100891 (PMC11540510; doi:10.7150/jca.100891)
Supplement: Supplementary file 1 — Supplementary tables. [file jcav15p6273s1.zip › Table S2.docx]

**Table S2. Clinical information for patients with pancreatic cancer from the First Affiliated Hospital of Dalian Medical University.**

| Characteristics |  | Number | Percentage (%) |
| --- | --- | --- | --- |
| Age (years) | <=65 | 30 | 55.6 |
|  | >65 | 24 | 44.4 |
| Gender | Male | 35 | 64.8 |
|  | Female | 19 | 35.2 |
| Clinical stage | Stage I | 20 | 38.5 |
|  | Stage II | 27 | 51.9 |
|  | Stage III | 1 | 1.9 |
|  | Stage IV | 4 | 7.7 |
| Pathological grade | G1-2 | 26 | 53.1 |
|  | G3-4 | 23 | 46.9 |
| Vital status | Survival | 11 | 22.0 |
|  | Death | 39 | 78.0 |
